# Unraveling *Burkholderia cenocepacia* H111 fitness determinants using two animal models

**DOI:** 10.1128/msystems.01354-24

**Published:** 2025-03-19

**Authors:** Sarah Paszti, Stefano Gualdi, Marta Torres, Luis Augusto, Freya Harrison, Leo Eberl

**Affiliations:** 1 Department of Plant and Microbial Biology, University of Zurich 30975 , Zürich, Switzerland; 2 Institute for Integrative Biology of the Cell (I2BC), University Paris-Saclay, CEA, CNRS, Gif-sur-Yvette, France; 3 School of Life Sciences, Gibbet Hill Campus, University of Warwick 117213 , Coventry, United Kingdom; University of Wisconsin-Madison, Madison, Wisconsin, USA

**Keywords:** *Burkholderia*, transposon sequencing, fitness determinants, Tnseq, host colonization, animal models, lipopolysaccharide, host bacteria interactions

## Abstract

*Burkholderia cenocepacia* is an opportunistic pathogen that has been associated with nosocomial outbreaks in hospitals and can cause severe respiratory infections among immunocompromised patients and individuals suffering from cystic fibrosis. The transmissibility and intrinsic antibiotic resistance of *B. cenocepacia* pose a significant challenge in healthcare settings. In this study, with the aim to identify novel drug targets to fight *B. cenocepacia* infections, we employed a genome-wide transposon sequencing (Tn-seq) approach to unravel fitness determinants required for survival in *Galleria mellonella* (*in vivo* infection model) and pig lung tissue (*ex vivo* organ model). A total of 698 and 117 fitness genes were identified for each of the models, respectively, and 62 genes were found to be important for both. To confirm our results, we constructed individual mutants in selected genes and validated their fitness in the two models. Among the various determinants identified was a rare genomic island (*I35_RS03700–I35_RS03770*) involved in O-antigen and lipopolysaccharide synthesis. We demonstrate that this gene cluster is required for virulence in the *G. mellonella* infection model but, by contrast, counteracts efficient colonization of pig lung tissue. Our results highlight the power of the Tn-seq approach to unravel fitness determinants that could be used as therapeutic targets in the future and show that the choice of the infection model for mutant selection is paramount.

**IMPORTANCE:**

The opportunistic pathogen *Burkholderia cenocepacia* has been associated with nosocomial infections in healthcare facilities, where it can cause outbreaks involving infections of the bloodstream, respiratory tract, and urinary tract as well as severe complications in immunocompromised patients. With the aim to identify novel targets to fight *B. cenocepacia* infections, we have used a genome-wide approach to unravel fitness genes required for host colonization in a clinical strain, *B. cenocepacia* H111. Among the various determinants that we identified is a rare genomic island that modifies the bacterial lipopolysaccharide. Our results highlight the power of the transposon sequencing approach to identify new targets for infection treatment and show the importance of using different infection models.

## INTRODUCTION

The opportunistic pathogen *Burkholderia cenocepacia* is a member of a metabolically diverse group of bacteria termed the *Burkholderia cepacia* complex (*Bcc*), which currently comprises more than 25 species (1, 2). *Bcc* species are commonly found in soil, plant rhizosphere, groundwater, industrial facilities (1, 3–6), and hospitals. In the latter, outbreaks frequently occur through exposure to contaminated solutions or medical instrumentation such as catheters or ventilators (7–11). They also exhibit resilience in antiseptic solutions and moist surfaces, among others, which allows them to persist for extended periods in healthcare settings. Some members of the *Bcc*, notably *B. cenocepacia* and *Burkholderia multivorans*, are particularly frequently associated with outbreaks in intensive care units. They are mainly associated with respiratory tract infections, especially in immunocompromised individuals and cystic fibrosis (CF) patients, but they can also cause bloodstream, skin, soft tissue, and urinary tract infections (12–14).

*B. cenocepacia* is able to form biofilms, resist oxidative stress, persist within macrophages, and produce a wide range of virulence factors, such as toxins, proteases, and type 6 secretion systems, all of which contribute to the clinical relevance of this organism (15). *B. cenocepacia* H111, originally isolated from a CF sputum sample (16, 17), has been established as a model to study pathogenicity, biofilm formation, and quorum sensing (QS) (12, 13, 18–21). It has been shown that this strain produces minor lung inflammation when colonizing healthy hosts (12). In strain H111, the production of various virulence factors and the ability to persist in a broad range of environments have been attributed to its large multireplicon genome (7.7 Mb), which encodes approximately 7,000 proteins (22).

In the last decade, new drugs and gene-editing therapies (e.g., clustered regularly interspaced short palindromic repeats [CRISPR]) have been developed to target the defective protein (i.e., the CF transmembrane regulator) responsible for the disease in the CF patients (23, 24). They contribute to an increase in the life expectancy and quality of life of patients. Despite these advances, the epidemiology of *B. cenocepacia* is not limited to CF patients, and nosocomial infections are an emerging challenge in healthcare settings (10, 25), where an increase in resistance and the emergence of acquired beta-lactamases among clinical isolates are observed (11). With the aim to identify novel targets for the treatment of *Bcc* infections, a better knowledge of the underlying mechanisms involved in pathogenicity and survival within hosts is needed.

Over the past few years, diverse systems have been used to study the genetic and phenotypic characteristics that confer bacterial persistence and virulence. Non-mammalian infection models have been established as attractive alternatives to traditional animal models (i.e., mice, rats) because of their practical advantages. They are cheap, easily maintained, high throughput, and reproducible (26). Examples of non-mammalian hosts include *Galleria mellonella*, *Caenorhabditis elegans*, *Drosophila melanogaster,* or *Danio rerio,* and they have proved to be very useful for evaluating the pathogenicity of *Bcc* strains (12, 19, 20, 27–30). For instance, the *G. mellonella* (greater wax moth or honeycomb moth) model is widely used due to its innate immunity being very similar to the immunity of mammals (31), and it has been demonstrated that it is a more accurate model for *Bcc* than other organisms such as *C. elegans* (29). Another alternative to live mammalian models is *ex vivo* models, which usually employ tissues or organs extracted from humans or animals. Their main advantage is that the spatial surface and the biochemical composition of the tissue are anatomically comparable to those in humans. One example is the *ex vivo* pig lung (EVPL) model (32), which comprises sections of pig bronchiole tissue incubated in synthetic sputum to mimic the etiology of the CF infection (33, 34).

One powerful approach to unravel the genetic determinants of fitness in a certain condition (e.g., host colonization) is transposon insertion sequencing (Tn-seq), consisting of the creation of a high-density transposon mutant library followed by challenging the library in a selective condition (e.g., host) and assessing the mutant abundance after a period of time (35–38). Underrepresented mutants after growth in the selective condition are indicative of genes that provide a fitness benefit to the bacterium. These genetic determinants of successful colonization or persistence, usually called “important” genes, are required in the tested condition, and thus, respective mutants will have a lowered fitness compared to the wild-type. On the contrary, mutants that are enriched under selective conditions are called “detrimental” genes, and their inactivation leads to increased fitness relative to the wild-type (39). This genome-wide method has been extensively used to identify genes required by pathogenic and beneficial bacteria (39) for survival in various hosts, including the non-mammalian infection hosts *G. mellonella* and *C. elegans* (40–42). Fitness genes identified in these screenings are promising targets to reduce host colonization. For instance, blocking some of the fitness pathways identified by Tn-seq in the plant pathogen *Agrobacterium tumefaciens* with chemical compounds resulted in reduced colonization and virulence (43). One important aspect of targeting such fitness determinants is that the risk of resistance development appears to be reduced relative to conventional antibiotics, as the Tn-Seq workflow cannot identify essential genes, and thus, there is no particular pressure for mutation of fitness genes (39, 44).

In this study, with the aim to unravel novel pathways that could be targeted in the future to reduce the colonization fitness of *B. cenocepacia* H111, we used the Tn-seq approach and two animal models, one *in vivo* (*G. mellonella*) and one *ex vivo* (EVPL). These animal host models address different questions, with *Galleria* simulating acute infection and the EVPL model mimicking a chronic infection. Both are widely used to assess pathogenicity and are thought to simulate the immune response and tissue anatomy encountered in patients. In our screening, we identified 698 and 117 genes that were important for survival in *G. mellonella* and EVPL, respectively. Of these, 62 genes were shared among both models, and we propose them as targets for reducing *B. cenocepacia* H111 colonization fitness. To confirm the obtained Tn-seq results, we constructed mutants in some of the identified genes, characterized them phenotypically, and evaluated their fitness in each of the models. Among the various factors identified was a rare genomic island in *B. cenocepacia* H111 involved in lipopolysaccharide (LPS) synthesis. Interestingly, while this LPS cluster is required for virulence in the *G. mellonella* infection model, it is detrimental for colonization of the EVPL, highlighting the importance of the host model for the identification of virulence factors that may serve as targets for the development of novel antimicrobials.

## MATERIALS AND METHODS

### Bacterial strains and culture conditions

Bacterial strains, primers, and plasmids used throughout this study are listed in Table S1. Strains were routinely grown in lysogeny broth (LB) (45), *Pseudomonas* isolation agar (PIA; Difco), or artificial sputum medium (SCFM1) (46) without glucose. Incubation was conducted at 37°C, either with shaking at 200 rpm–220 rpm under aerobic conditions or statically. The Tn*23 B. cenocepacia* H111 transposon library was previously constructed by our group (47). When growing the library, and to ensure expression of downstream genes, the media was supplemented with L-rhamnose 0.2% (wt/vol), as the transposon used for constructing the library carries a rhamnose-inducible promoter reading outward.

When required, appropriate antibiotics were supplemented using the following concentrations: chloramphenicol (Cm) 80 µg/mL, trimethoprim (Tp) 50 µg/mL, gentamicin (Gm) 20 µg/mL, and polymyxin B sulfate (PMB) 25 µg/mL for *B. cenocepacia* H111; kanamycin 25 µg/mL, Cm 20 µg/mL, Tp 50 µg/mL, and Gm 20 µg/mL for *Escherichia coli* strains.

### Tn-seq experimental setup

**Fig 1:**
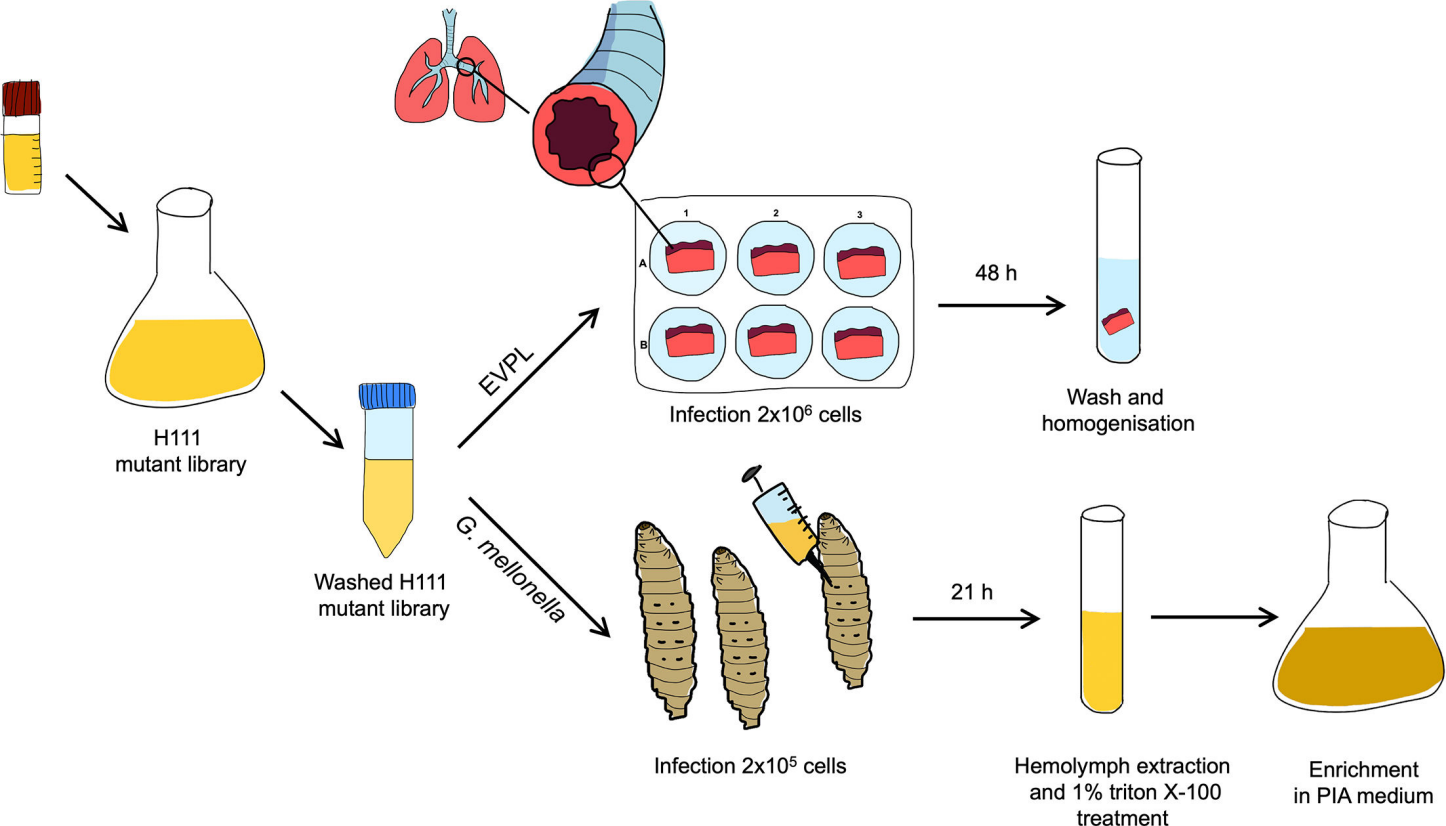
Experimental overview of the identification of host-associated fitness determinants in *B. cenocepacia* H111 in the *G. mellonella* and the EVPL models using a Tn-seq approach. For more detailed information, please refer to Materials and Methods.

The growth of Tn*23 B. cenocepacia* H111 transposon library (47) for the assays in *G. mellonella* and EVPL was conducted as follows (Fig. 1). Briefly, one aliquot of the library was thawed and washed twice using the corresponding fresh medium, i.e., LB for *G. mellonella* and SCFM1 for EVPL. The washed pellet was resuspended in 1.5 mL fresh medium and transferred into a tube containing 8.5 mL of LB or SCFM1 medium supplemented with a final concentration of 0.2% (wt/vol) L-rhamnose, incubated for 1 h at 37°C without shaking, then all transferred to 15 mL of fresh LB or SCFM1 amended with 0.2% (wt/vol) L-rhamnose to obtain 25 mL. For the EVPL, PMB 25 µg/mL was added as well. Cells (starting OD_600_ 0.02) were grown to OD_600_ 0.8–1. Then, transposon mutant cells were washed by centrifugation for 10 min at 4,000 rpm and used in the different models, as explained below.

For the *G. mellonella* model, the injection of the larvae (two replicates, *n* = 50 each, named Tn1 and Tn2) was performed as described previously (48). Larvae were obtained from fishing shops in Switzerland, either in Zurich (Reptile Food GmbH) or Basel (Bait Express GmbH). Transposon mutant cells were washed and resuspended in phosphate-buffered saline (PBS). The OD_600_ was adjusted to 0.0625, and 10 µL (2 × 10^5^ cells) was injected into the last left proleg of each *G. mellonella* larvae using a 27 × 3/4 gauge needle (BD Microlance, Waltham, MA, USA) and a Hamilton syringe (Sigma-Aldrich, USA). After 21 h of incubation at 30°C, the hemolymph of each *G. mellonella* was extracted as follows. The last abdominal segment of each larva was removed with a sterile blade. Then, the 50 larvae in each group (Tn1 and Tn2) were squeezed with the help of tweezers, and their hemolymph (20 µL–70 µL per larva) was collected into a tube containing 15 mL of PBS supplemented with 1% (vol/vol) Triton X-100 to remove eukaryotic cells, as described by Bruchmann et al. (41). The tubes were centrifuged at 250 × *g* for 5 min, the pellet discarded, and the supernatant kept and centrifuged at 4,000 × *g* for 10 min. After removal of the supernatant, the bacterial cells were resuspended in 25 mL of liquid PIA and enriched until OD_600_ 1. The cells were then pelleted and stored at −20°C for further genomic extraction.

For the EVPL model, the preparation of lung material was done as described in Harrison et al. (33, 34) with some modifications. Briefly, pig lungs were collected from the butcher and dissected using a sterile razor blade. Five square millimeter sections of bronchiole tissue were cut and washed three times with cell culture medium, consisting of 1:1 mix of RPMI-1640 medium (Sigma-Aldrich, USA) and Dulbecco’s modified Eagle medium (Sigma-Aldrich, USA). A fourth wash was conducted with SCFM1. Sterile 24-well plates were prepared by aliquoting 400 µL of SCFM1 supplemented with 0.8% (wt/vol) agarose, 0.2% (wt/vol) L-rhamnose, and 25 µg/mL of PMB into each well. Bronchiole sections were placed on the surface of the medium once solidified. Transposon mutant cells were washed and resuspended in SCFM1. OD_600_ was adjusted to 0.02. Each lung section (*n* = 36) was immersed in 600 µL of the bacterial suspension in SCFM1 supplemented with 25 µg/mL of PMB. Well plates were covered with a gas-permeable membrane (Sigma-Aldrich, USA) and incubated at 37°C for 48 h without shaking. After incubation, bronchiole sections were washed in PBS to remove planktonic cells and transferred into reinforced tubes with 1 g of 2.4 mm metal beads (Fisherbrand, cat. no. 15505809). Each tissue section was homogenized using a Fast-Prep-24 5G instrument (MP Biomedicals, USA) at 4 m/s for 40 s. The suspensions corresponding to the 36 different bronchiole sections were pooled together in a 50 mL tube for genomic extraction.

### Genomic DNA preparation for sequencing

For genomic DNA extraction, the GeneElute Bacterial Genomic DNA kit (Sigma-Aldrich, USA, ref: NA2110) was used following the manufacturer’s instructions. Then, the DNA samples were prepared for Illumina sequencing using the circle method (49, 50) as detailed by Vitale et al. (51). Analysis of the sequenced DNA libraries was performed as previously described with some modifications (48, 51). Sequencing files were trimmed for adaptors, and the transposon sites were mapped against the *B. cenocepacia* H111 genome using Tn-seq Explorer (52). To facilitate comparison with other studies, locus tags (e.g., *BCALXXXX, BCAMXXXX*) and gene symbols of orthologs in the closely related *B. cenocepacia* J2315 (the type strain of the species [53]) are used throughout this work (Table S2). In cases where no ortholog is found in J2315, H111 identifiers (i.e., *I35_RSXXXXX*) are given.

Unique insertion density (UID) was calculated for each gene by dividing the unique transposon insertion counts (UICs) within a gene by the gene length. To avoid calculation errors when UIC = 0, UIC + 0.01 was used. After obtaining the UID, it was normalized (NUID) by the total UIC in each sequencing sample. Fitness values were calculated as the log_2_ fold change (FC) of each gene, for which the NUID of the gene in the condition sample (i.e., *G. mellonella* or EVPL) was divided by the NUID of the gene in the corresponding control, which was the *B. cenocepacia* H111 Tn*23* library grown in LB (47). The difference between the NUID in the tested condition and the control is called dNUID. A gene with negative fitness (FC ≤ −1 and dNUID ≥ 0.004) means that the mutant in that gene is less abundant in that given condition compared with the control. These are called “important” genes that, when mutated, result in reduced colonization ability (i.e., reduced fitness). A gene with a positive fitness (FC ≥ 1 and dNUID ≤ −0.004) means that the mutant in that gene is more abundant in that condition compared to the control. These are called “detrimental” genes that, when mutated, result in improved colonization ability (i.e., improved fitness). Cluster of orthologous groups (COG) was added using EggNOG version 4.5 (54).

### Construction of *B. cenocepacia* H111 mutant strains

To validate the Tn-seq data and confirm the results obtained, individual mutants were constructed. Insertion mutagenesis was performed using the suicide plasmid pSHAFT2 (55) on a set of selected genes: *BCAL1700, BCAL1994, BCAL2403–BCAL2406* (this mutant will be referred to as *BCAL2406,* in which the downstream genes *BCAL2403–BCAL2405* are mutated as well), *BCAL2370, BCAM2231, BCAM2224, BCAL2981,* and *BCAM2739*. For each mutation, an internal fragment of the target gene was amplified using high-fidelity Phusion polymerase. A list of the oligonucleotides used can be found in Table S1. After ligating the amplified region into the pSHAFT2 vector, the plasmid containing the right region was transformed into competent *E. coli* CC118λpir cells. Integration of the plasmids into *B. cenocepacia* H111 wild-type occurred via triparental mating. The conjugants were selected on PIA containing Cm 80 µg/mL and were confirmed by PCR by amplifying fragments flanking both sides of the integrated plasmid. In-frame deletion mutants were created in selected genes (*BCAL0399*, *I35_RS03700–I35_RS03770*) as described by Flannagan et al. (56). Fragments flanking the gene of interest were amplified by PCR and cloned into the suicide plasmid pGPI-*SceI* (56) that carries a *SceI* recognition site. The constructed plasmid was transferred to *B. cenocepacia* H111 by triparental mating, and the conjugants were selected on PIA containing Tp 50 µg/mL. The integration was confirmed by PCR. Plasmid pDAI (56) encoding the *SceI* restriction enzyme was then transferred through conjugation into the *B. cenocepacia* H111 derivative carrying pGPI-*SceI* integrated in its genome. Correct deletion was confirmed by PCR amplification and sequencing.

### Validation of individual mutants in *G. mellonella* and EVPL

Overnight cultures in LB of the mutants and the wild-type strain were sub-cultured in 20 mL LB (in 100 mL Erlenmeyer flask). Before reaching OD_600_ = 1, the cells were washed by centrifugation for 10 min at 4,000 rpm and resuspended in fresh media. The injection of *G. mellonella* was performed as described above. Larvae injected with PBS were used as negative control. Survival was monitored daily over 72 h at 30°C. Regarding the EVPL, bronchiole cubes were prepared as explained before, and each piece was immersed with 600 µL of a bacterial suspension (OD_600_ = 0.02, 2 × 10^6^ cells per well) in SCFM1. Sterile SCFM1 was used as a negative control. After 48 h of incubation, bronchiole sections were washed and homogenized, and colony-forming units (CFUs) determinations were conducted on PIA plates.

### Phenotypic characterization of the constructed mutants

To evaluate some phenotypes of the individual mutants constructed, overnight cultures of the wild-type and mutant strains were prepared and washed, and their OD_600_ was adjusted to 1. Colony size was assessed after conducting dilutions and spotting on LB medium. Colony morphology was assessed in nutrient broth-yeast extract-glycerol (NYG) media (57). Congo red (CR) binding was evaluated in yeast extract-mannitol (YEM) media supplemented with 80 µg/mL CR. Exopolysaccharide (EPS) production was assessed on modified YEM medium with 0.06% (wt/vol) yeast extract (58) using two methods: cross-colonies (59) and in plates that were incubated vertically after spotting and drying the different strains. Swimming motility assays were performed as previously described (18, 48) using LB supplemented with 0.2% (wt/vol) agar. PMB sensitivity assay was conducted on 1.5% (wt/vol) Mueller-Hinton II cation-adjusted agar medium supplemented with 64, 128, 256, and 512 µg/mL PMB. All the plates were incubated at 37°C for 4 days, except motility plates that were observed and measured after 2 days and PMB assay that was measured at 24 h. All the assays were conducted in triplicate. Bacterial cell morphology was observed by phase contrast using a Leica DM6000 microscope.

For LPS characterization, bacterial cultures were grown in 100 mL of LB to an OD_600_ ±2. Thereafter, LPS was isolated by using the phenol:water method of Westphal and Jann (60). In brief, the bacterial pellet was mixed into a 50% (vol/vol) phenol:water solution and incubated at 65°C for 15 min while being stirred. From the cooled water phase, insoluble material was removed by centrifugation. To get rid of the phenol, the clear extract was dialyzed under running tap water and thereafter dialyzed against distilled water. The samples were then lyophilized. SDS-PAGE was used for the LPS analysis, in which samples (2 µg of LPS per sample) were loaded onto a 15% (wt/vol) polyacrylamide separation gels layered with 4% (wt/vol) polyacrylamide stacking gel, using standard electrophoresis buffers and migration conditions (61). The silver nitrate method was used to stain the gels (62). For the lipid A preparation, the triethylamine-citrate method was used (63). In brief, LPS samples were suspended at a concentration of 10 µg/µL in a 0.01 M triethylamine-citrate solution (1:1 molar ratio, pH 3.6) and incubated for 1 h at 100°C. Thereafter, samples were lyophilized and suspended in methanol. Using a mixture of chloroform:methanol:water (3:1.5:0.25, vol:vol:vol) at a concentration of 10 µg/µL, lipid A fractions were extracted after centrifugation at 7,000 × *g* for 10 min at 4°C. Using an AXIMA performance (Shimadzu Biotech, Japan) matrix-assisted laser desorption ionization-time of flight (MALDI-TOF) mass spectrometer, the molecular species present in the lipid A fraction were analyzed. One microliter of a lipid A suspension (1 µg/µL) in chloroform:methanol:water (3:1.5:0.25, vol:vol:vol) was deposited, mixed with 1 µL of a 2,5-dihydroxybenzoic acid matrix suspended at 10 µg/µL in the same solvent, and then dried. To desorb the analyte ions from the matrix, pulses from a 337 nm nitrogen laser were used. Spectra were obtained in the negative-ion mode at 20 kV, with the linear detector. A peptide mass standard kit (AB SCIEX, Canada) was used for mass calibration.

## RESULTS

### Genome-wide profiling of *B. cenocepacia* H111 fitness genes for host colonization

In this study, a *B. cenocepacia* H111 Tn*23* transposon library was used (47) for the identification of fitness factors in *G. mellonella* and the EVPL model. In brief, *G. mellonella* larvae were injected (2 × 10^5^ cells per larvae), and after 21 h, the hemolymph was extracted. After the removal of eukaryotic cells, the hemolymph was inoculated into selective PIA medium to enrich the recovered transposon mutants (i.e., to ensure that enough material is available for sequencing and to prevent growth of bacteria residing within the larvae) (Fig. 1). In the EVPL model, pieces of pig lung bronchiole tissue were submerged for 48 h into a suspension containing the *B. cenocepacia* H111 Tn*23* transposon library (2 × 10^6^ cells per well) in SCFM1.

**Fig 2:**
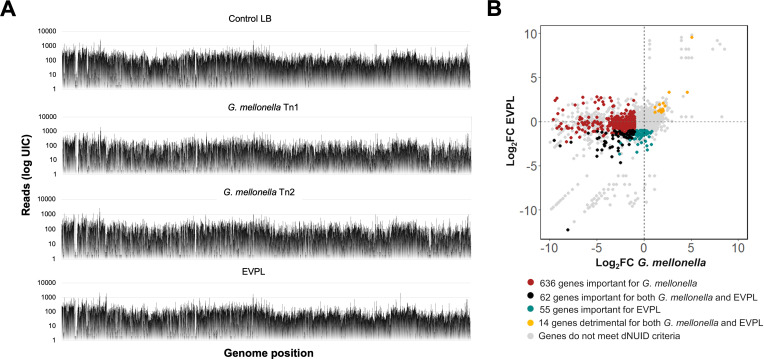
Fitness determinants identified in *B. cenocepacia* H111 using the *G. mellonella* and EVPL models. (A) Distribution of mutants across the *G. mellonella* (replicates Tn1 and Tn2) and EVPL samples; *x*-axis represents the position in *B. cenocepacia* H111 genome, and *y*-axis represents log of number of reads (UICs). (B) Biplot showing the fitness values (FC) for each of the two models. The 636 important genes uniquely needed for survival in *G. mellonella* are shown in red, the 55 important fitness determinants involved uniquely in the colonization in the EVPL model are shown in turquoise, the 62 core important fitness determinants shared for the two models are indicated in black, and the 14 core detrimental fitness determinants shared for the two models are shown in yellow.

It is important to keep in mind that Tn-seq approaches employing infection models may be biased due to bottlenecks arising from too small infection doses. As a consequence of the reduced genetic diversity of the infection inoculum, some insertion mutants will be lost and would erroneously be scored as a fitness determinant (39, 64). However, sequence analysis of our Tn-seq samples did not show any particular enrichment of a few transposon insertions, as would be expected in the case of a bottleneck (Fig. 2A; Table S2). We used these data to determine fitness values for each gene by comparing the transposon insertion frequencies between each test sample (*G. mellonella* or EVPL) and a control (LB)(47) using the following parameters: FC ≤ −1 and dNUID ≥ 0.004 for important genes and FC ≥ 1 and dNUID ≤ −0.004 for detrimental genes. A total of 698 and 117 genes were identified to be important for the fitness in *G. mellonella* and the EVPL model, respectively (Table S2). Regarding the detrimental genes, a total of 197 and 86 genes were identified in each model, respectively (Table S2). In this manuscript, we will mainly focus on important genes (i.e., FC ≤ −1 and dNUID ≥ 0.004), since they represent more attractive targets for the development of therapeutics aiming to reduce the colonization fitness of the pathogen.

Clusters of orthologous groups classification of the 698 identified fitness genes in *G. mellonella* revealed that the most abundant COG categories (apart from class S, function unknown) are: K (transcription), E (amino acid transport and metabolism), M (cell wall/membrane/envelope biogenesis), C (energy production and conversion), P (inorganic ion transport and metabolism), and H (coenzyme transport and metabolism). In class K, there are several LysR, AraC, and TetR family transcriptional regulators. To class E belong genes involved in aromatic amino acid biosynthesis such as the anthranilate synthase subunit II (*BCAL0398, trpG*) or a shikimate kinase (*BCAL0279, aroK*). In class C, we found genes related to the tricarboxylic acid cycle, such as succinyl-CoA synthetase subunit alpha (*BCAL0957, sucD*). In class P, we identified, for instance, an ornibactin receptor (*BCAL1700, orbA*). In class H, we found genes related to molybdenum cofactor biosynthesis (*BCAL0962, moaC; BCAL1923, moaD*). Finally, in class M, we identified a poorly annotated 15-gene cluster, spanning the region from a hypothetical protein (*I35_RS03700*) to a glycosyl transferase (*I35_RS03770*).

With regard to the EVPL model, apart from genes belonging to class S (function unknown), most of the 117 genes belonged to COG categories E, H, M, and K. In class E, we found genes related to acetolactate synthesis (*BCAL2358 = ilvH*, *BCAL2359, ilvI*) and tryptophan synthesis (*BCAM0991, trpB; BCAM0993, trpA*). In category H, we found genes involved in the synthesis of cofactors such as NAD(+) (*BCAL2716–BCAL2718, nadBCA*). Finally, among the genes in category M, we find a rod shape-determining gene (*BCAL0480*) and a large-conductance mechanosensitive channel (*BCAL2024*).

### Identification of host fitness genes in *B. cenocepacia* H111

#### Core host fitness genes

A total of 698 and 117 fitness determinants were identified as important genes (FC ≤ −1 and dNUID ≥ 0.004) for *G. mellonella* and EVPL, respectively (Table S2). Of these, 62 genes were shared between the two hosts (Fig. 2B; Table 1). We will hereafter refer to them as “core host fitness genes.” This would mean that 636 and 55 genes would be unique for survival in *G. mellonella* and EVPL, respectively.

**TABLE 1:** *Burkholderia cenocepacia* H111 core fitness genes in the *Galleria mellonella* and the EVPL models^*a*^

H111 locus tag	H111 description	COG	J2315 ortholog	J2315 description	J2315 gene symbol	Avg FC: *Galleria*	Avg dNU ID: *Galleria*	FC: EVPL	dNUID: EVPL
Important genes (FC ≤ −1)									
*I35_RS02535*	Glyceraldehyde-3-phosphate dehydrogenase	C	BCAL3388	Glyceraldehyde 3-phosphate dehydrogenase 1	gapA	−4.2282	0.005	−2.5747	0.0044
*I35_RS01410*	3-Dehydroquinate synthase	E	BCAL0280	3-Dehydroquinate synthase	aroB	−3.3733	0.0155	−2.8273	0.0151
*I35_RS01570*	Histidinol dehydrogenase	E	BCAL0312	Histidinol dehydrogenase	hisD	−4.4539	0.0199	−1.2172	0.012
*I35_RS01580*	Imidazoleglycerol-phosphate dehydratase	E	BCAL0314	Imidazoleglycerol-phosphate dehydratase	hisB	−1.9126	0.0172	−1.7608	0.0167
*I35_RS01590*	Imidazole glycerol phosphate synthase subunit HisH	E	BCAL0316	Imidazole glycerol phosphate synthase subunit HisH	hisH	−1.6323	0.0164	−1.5091	0.0163
*I35_RS01595*	1-(5-Phosphoribosyl)-5-[(5-phosphoribosylamino)methylideneamino] imidazole-4-carboxamide isomerase	E	BCAL0317	1-(5-phosphoribosyl)-5-[(5-phosphoribosylamino)methylideneamino] imidazole-4-carboxamide isomerase	hisA	−1.884	0.0122	−1.7925	0.0131
*I35_RS01600*	Imidazole glycerol phosphate synthase subunit HisF	E	BCAL0318	Imidazole glycerol phosphate synthase subunit HisF	hisF	−3.3092	0.0212	−1.9002	0.0173
*I35_RS01610*	Phosphoribosyl-ATP pyrophosphatase	E	BCAL0320	Phosphoribosyl-ATP pyrophosphatase	hisE	−2.0967	0.0144	−1.4308	0.0119
*I35_RS03040*	Phospho-2-dehydro-3-deoxyheptonate aldolase	E	BCAL3282	Phospho-2-dehydro-3-deoxyheptonate aldolase	aroG	−1.2777	0.0171	−1.848	0.021
*I35_RS04575*	3-Phosphoshikimate 1-carboxyvinyltransferase	E	BCAL2952	3-Phosphoshikimate 1-carboxyvinyltransferase	aroA	−8.8973		−2.7192	0.0071
*I35_RS05290*	N-succinylarginine dihydrolase	E	BCAL1063	Succinylarginine dihydrolase	astB	−1.2787	0.0046	−1.4061	0.0049
*I35_RS06780*	Chorismate synthase	E	BCAL1467	Chorismate synthase	aroC	−3.5897	0.0086	−2.959	0.0082
*I35_RS08950*	ATP phosphoribosyltransferase regulatory subunit	E	BCAL1874	ATP phosphoribosyltransferase regulatory subunit	hisZ	−1.6931	0.0086	−1.8542	0.0091
*I35_RS09215*	Aminotransferase	E	BCAL1927	Aminotransferase AlaT		−4.0519	0.0082	−2.3784	0.0072
*I35_RS11440*	Acetolactate synthase	E	BCAL2358	Acetolactate synthase 3 regulatory subunit	ilvH	−2.6825	0.0076	−2.2065	0.0074
*I35_RS33445*	Threonine transporter RhtB	E				−1.1048	0.0045	−1.2637	0.005
*I35_RS01995*	Anthranilate synthase subunit II	EH	BCAL0398	Anthranilate synthase component II	trpG	−4.963	0.0315	−1.9146	0.0241
*I35_RS20440*	ABC transporter substrate-binding protein	ET	BCAM0874	Periplasmic solute-binding protein		−2.2995	0.0062	−1.4465	0.0051
*I35_RS01405*	Shikimate kinase	F	BCAL0279	Shikimate kinase	aroK	−1.4705	0.015	−1.785	0.0172
*I35_RS01565*	ATP phosphoribosyltransferase	F	BCAL0311	ATP phosphoribosyltransferase catalytic subunit	hisG	−1.8023	0.0196	−1.4586	0.0181
*I35_RS14635*	2,3-Bisphosphoglycerate-dependent phosphoglycerate mutase	F	BCAL0739	Phosphoglyceromutase	gpmA	−2.7635	0.0117	−2.9903	0.0124
*I35_RS02350*	Sugar kinase	G	BCAL3425	Putative sugar kinase		−1.5601	0.0189	−1.0678	0.0155
*I35_RS05160*	Trehalose-phosphatase	G	BCAL1035	Putative trehalose-phosphatase	otsB	−3.1343	0.0088	−1.0443	0.006
*I35_RS01865*	3-Octaprenyl-4-hydroxybenzoate carboxy-lyase	H	BCAL0371	3-Octaprenyl-4-hydroxybenzoate carboxy-lyase		−1.4385	0.0078	−1.7685	0.0103
*I35_RS04070*	3-Octaprenyl-4-hydroxybenzoate carboxy-lyase	H	BCAL3058	3-Octaprenyl-4-hydroxybenzoate carboxy-lyase	ubiD	−4.7651	0.0072	−1.8159	0.0054
*I35_RS09970*	Phosphoenolpyruvate synthase	H	BCAL2074	Phosphoenolpyruvate synthase	ppsA	−9.5316	0.0061	−2.0998	0.0047
*I35_RS11435*	Ketol-acid reductoisomerase	H	BCAL2357	Ketol-acid reductoisomerase	ilvC	−3.4049	0.0123	−3.9767	0.0129
*I35_RS11445*	Acetolactate synthase	H	BCAL2359	Acetolactate synthase 3 catalytic subunit	ilvI	−2.7619	0.0105	−3.3043	0.0112
*I35_RS14645*	molybdopterin biosynthesis protein MoeB	H	BCAL0737	Putative molybdopterin biosynthesis protein		−2.52	0.0091	−1.4089	0.0071
*I35_RS14995*	adenosylmethionine-−8-amino-7-oxononanoate aminotransferase BioA	H	BCAL0667	Aadenosylmethionine-8-amino-7-oxononanoate transaminase	bioA	−4.4619	0.0079	−3.2152	0.0074
*I35_RS15005*	ATP-dependent dethiobiotin synthetase BioD	H	BCAL0665	Dithiobiotin synthetase	bioD	−4.2074	0.0069	−2.7463	0.0062
*I35_RS15010*	biotin synthase	H	BCAL0664	Biotin synthase	bioB	−5.0167	0.0112	−3.9231	0.0108
*I35_RS12905*	aminotransferase	I	BCAL2719	Putative transmembrane fatty acid desaturase		−10.2038	0.0114	−1.5124	0.0074
*I35_RS29975*	squalene--hopene cyclase	I	BCAM2831	Squalene-hopene cyclase	shc	−1.1763	0.0086	−1.4686	0.0099
*I35_RS03165*	tRNA dimethylallyltransferase	J	BCAL3257	tRNA delta(2)-isopentenylpyrophosphate transferase	miaA	−4.9429	0.007	−3.1924	0.0065
*I35_RS02970*	LysR family transcriptional regulator	K	BCAL3301	Oxidative stress regulatory protein	oxyR	−5.9141	0.0159	−1.2754	0.0095
*I35_RS18455*	AraC family transcriptional regulator	K	BCAM0481	AraC family regulatory protein		−1.2213	0.0041	−1.0003	0.0042
*I35_RS19165*	LuxR family transcriptional regulator	K	BCAM0623	Two-component regulatory system response regulator protein		−1.36	0.0057	−1.0316	0.005
*I35_RS01530*	Lipid asymmetry maintenance protein MlaA	M	BCAL0304	VacJ-like lipoprotein		−1.2261	0.0065	−1.1521	0.0065
*I35_RS03305*	Cell envelope biogenesis protein TonB	M	BCAL3202	Possible TolA-related transport transmembrane protein	tolA	−1.2603	0.0071	−1.3016	0.0073
*I35_RS03795*	UDP-glucose 4-epimerase	M	BCAL3117	UDP-glucose epimerase	galE	−4.9581	0.0118	−1.104	0.0065
*I35_RS11675*	Glycosyl transferase family 1	M	BCAL2403	Putative LPS core biosynthesis protein	wabP	−1.5839	0.009	−2.5726	0.0118
*I35_RS11680*	Glycosyl transferase	M	BCAL2404	Putative glycosyltransferase	wabQ	−1.6203	0.0085	−1.1009	0.007
*I35_RS14640*	Peptidase S41	M	BCAL0738	C-terminal processing protease-3		−3.3262	0.0073	−1.545	0.0054
*I35_RS01640*	2-Alkenal reductase	O	BCAL0326	Subfamily S1B serine peptidase		−1.4116	0.0122	−1.0188	0.0105
*I35_RS32135*	SAM-dependent methyltransferase	Q				−1.8285	0.0064	−1.3324	0.0058
*I35_RS02995*	YggS family pyridoxal phosphate enzyme	S	BCAL3291	Hypothetical protein		−2.2999	0.0066	−2.6332	0.0075
*I35_RS03175*	Membrane protein	S	BCAL3255	Putative DedA family protein		−7.7740	0.0091	−2.3205	0.0078
*I35_RS03805*	Glycosyl transferase	S	BCAL3115	Glycosyltransferase	wbxA	−4.1082	0.0065	−2.2902	0.0058
*I35_RS03810*	Teichoic acid transporter	S	BCAL3114	Putative O-antigen exporter	wzx	−8.0742	0.0041	−12.2408	0.0043
*I35_RS04330*	tRNA-Arg	S				−2.969	0.0103	−1.4434	0.0075
*I35_RS05475*	Hypothetical protein	S	BCAL1103	Putative OsmB-like lipoprotein		−2.3518	0.0062	−1.3198	0.0047
*I35_RS12120*	Hypothetical protein	S				−2.6121	0.007	−1.0834	0.0049
*I35_RS20565*	Hypothetical protein	S	BCAM0900	Hypothetical protein		−4.1907	0.0051	−2.2513	0.0042
*I35_RS21075*	Bacteriocin production protein	S	BCAM0997	Colicin V production protein		−2.9709	0.008	−1.1528	0.0052
*I35_RS21635*	GNAT family acetyltransferase	S	BCAM1185	Hypothetical protein		−4.0567	0.0075	−1.294	0.0048
*I35_RS29545*	Hopanoid biosynthesis associated radical SAM protein HpnH	S	BCAM2739	MoaA/NifB/PqqE family protein		−2.516	0.0055	−4.6388	0.0069
*I35_RS30095*	Membrane protein	S	BCAS0016	Hypothetical protein		−2.7518	0.0065	−1.6464	0.0056
*I35_RS33710*	Purine nucleoside phosphorylase	S	BCAS0579	Hypothetical protein		−2.8921	0.0064	−1.2115	0.0048
*I35_RS15820*	Chemotaxis protein CheY	T	BCAL0499	Two-component regulatory system, response regulator protein		−1.483	0.0054	−1.4236	0.0055
*I35_RS03310*	Biopolymer transporter ExbD	U	BCAL3201	Putative TolR-related protein	tolR	−2.097	0.0167	−1.7141	0.0152
*I35_RS03315*	Protein tolQ	U	BCAL3200	Putative TolQ transport transmembrane protein	tolQ	−1.0263	0.0106	−1.1587	0.0117
*I35_RS00865*	tRNA-Ala	S				1.7114	−0.0342	1.3301	−0.0212
Detrimental genes (FC ≥ 1)									
*I35_RS02065*	tRNA-Ile	S				1.1783	−0.0116	1.6301	−0.0186
*I35_RS02250*	tRNA-Pro	S				2.677	−0.0056	3.348	−0.0091
*I35_RS07210*	tRNA-Leu	S				2.2109	−0.0097	1.7726	−0.0063
*I35_RS07790*	Hypothetical protein	S	BCAL1645	Hypothetical protein		1.5221	−0.0084	1.2308	−0.006
*I35_RS08860*	Hypothetical protein	S	BCAL1857	Hypothetical protein		1.1529	−0.013	1.0736	−0.0074
*I35_RS09420*	N-acetyltransferase GCN5	S	BCAL1968	Putative acetyltransferase		1.842	−0.0152	1.0789	−0.0047
*I35_RS12330*	Nitrate ABC transporter ATP-binding protein	P				2.0291	−0.0065	2.0651	−0.0061
*I35_RS12755*	tRNA-Leu	S				4.5636	−0.0213	3.348	−0.0083
*I35_RS12885*	50S ribosomal protein L33	J	BCAL2715	50S ribosomal protein L33	rpmG	2.0157	−0.0104	1.2043	−0.0041
*I35_RS13625*	tRNA-Met	S				5.0872	−0.0041	9.5216	−0.0072
*I35_RS15385*	Lipoprotein	S	BCAL0588	Putative lipoprotein		1.9779	−0.0136	1.4372	−0.0078
*ncS03*	Small_RNA	S				1.6571	−0.0185	1.9297	−0.0184
*ncS27*	Small_RNA	S				1.9739	−0.0555	1.3303	−0.0262

^*a*^ FC and dNUID are shown.

**Fig 3:**
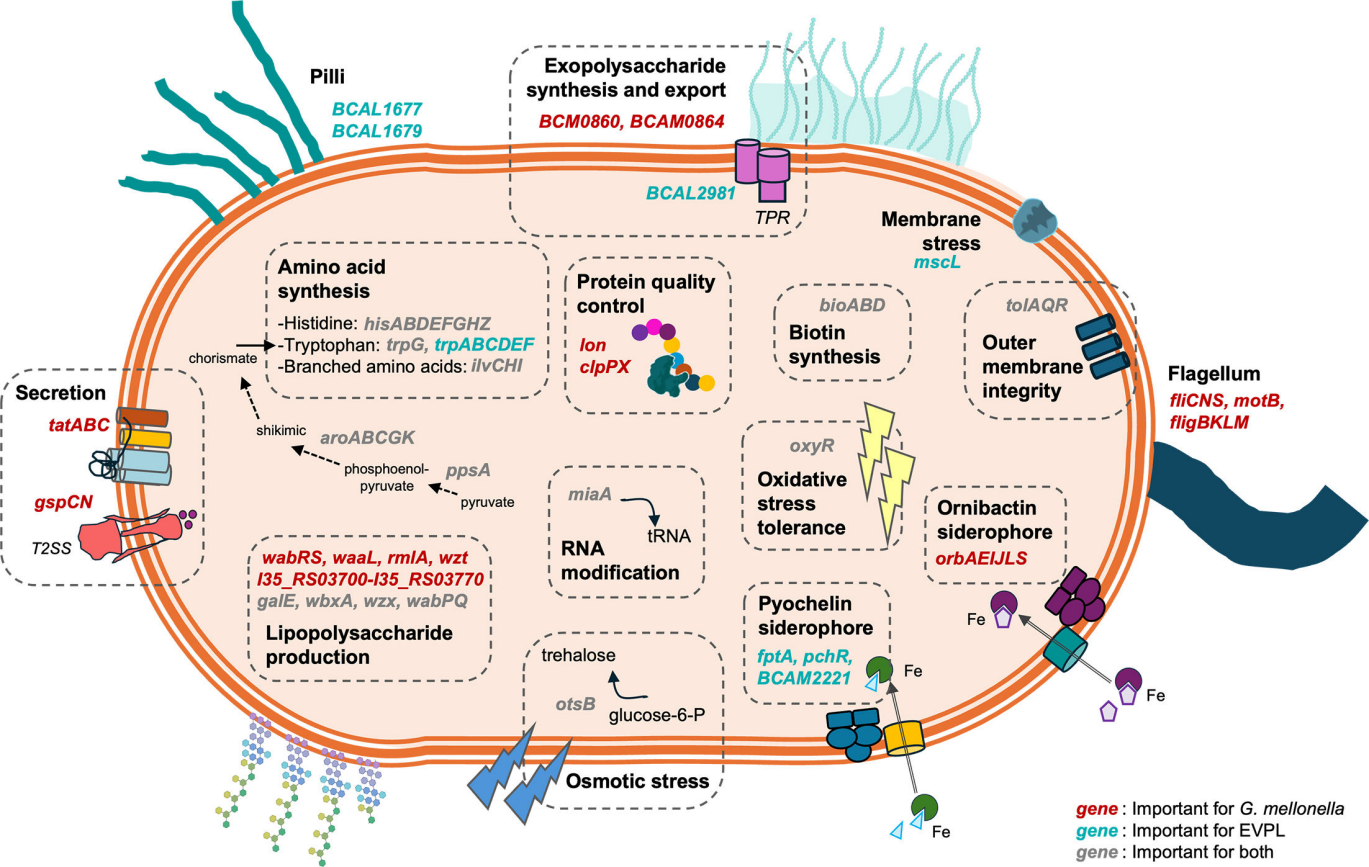
Simplified overview of *B. cenocepacia* H111 fitness determinants that are important for host colonization. Examples of genes uniquely important for survival in *G. mellonella* are shown in red, genes involved uniquely in the colonization of the EVPL model are shown in turquoise, and important fitness determinants shared for the two models are indicated in gray. Not all the fitness genes are represented. See Table S2 for a complete list.

Of the 62 “core important fitness genes” (Fig. 2B and 3; Table 1), approximately one-third are involved in amino acid transport or metabolism (COG class E), including genes encoding for histidine synthesis (*BCAL0311, hisG; BCAL0312, hisD; BCAL0314, hisB; BCAL0316*, *hisH; BCAL0318, hisF; BCAL0320, hisE*), the anthranilate synthase subunit II (*BCAL0398, trpG*), and other genes directing the biosynthesis of aromatic amino acids such as *aroKB* (*BCAL0279, BCAL0280*). Other interesting shared genes across the two hosts are coding for biotin biosynthesis (*BCAL0664, bioB; BCAL0665, bioD; BCAL0667, bioA*). Finally, other examples are genes related to COG class M and encode for LPS synthesis and protein glycosylation, including *BCAL2403–BCAL2404 (wabPQ*), *BCAL3114* (*wzx*), *BCAL3115* (*wbxA*) and *BCAL3117* (*galE*). Interestingly, *BCAL3114, BCAL3115,* and *BCAL3117* (recently re-annotated as *ogcX, ogcA,* and *ogcE*) are part of a gene cluster, which is required for the assembly and export of an undecaprenyl-linked trisaccharide that is incorporated into more than 100 proteins and is highly conserved in the *Burkholderia* genus. Mutants in these genes have previously been shown to affect the innate immune response of *G. mellonella*, likely due to the lack of glycosylated proteins (65, 66).

Of the 197 and 86 genes identified (FC ≥ 1 and dNUID ≤ −0.004) to be required for host fitness in *G. mellonella* and pig lung tissue, respectively, 14 genes were found to be positively selected in both models (Fig. 2B; Table 1), and these we refer to as “core detrimental fitness genes.” Most of them code for hypothetical proteins (*BCAL1645*, *BCAL1857*), small RNAs (*ncS03, ncS27*), and tRNAs (*I35_RS00865,* tRNA-Ala; *I35_RS02065,* tRNA-Ile; *I35_RS02250,* tRNA-Pro; *I35_RS07210,* tRNA-Leu; *I35_RS12755,* tRNA-Leu; *I35_RS13625,* tRNA-Met), and we hypothesize that they may have regulatory functions

#### Host-specific fitness genes

**Fig 4:**
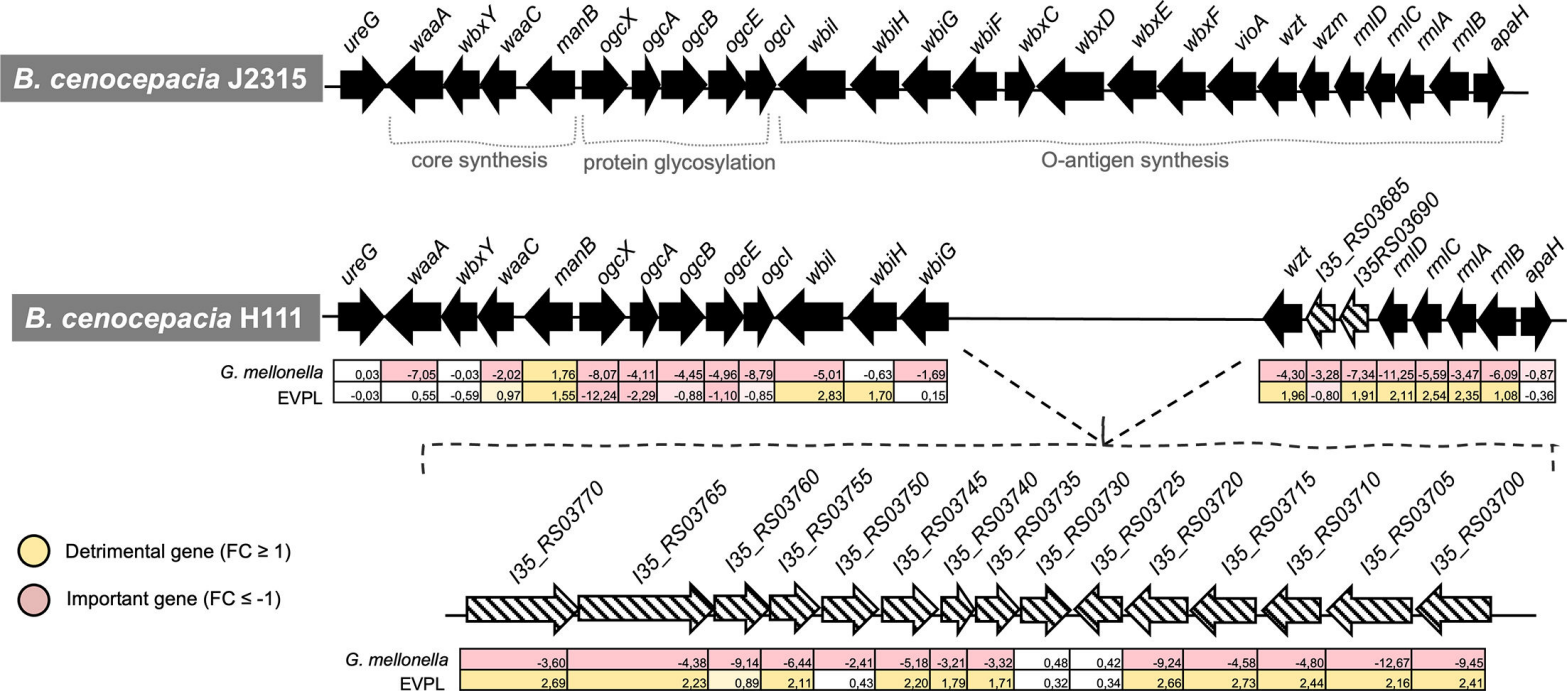
Gene overview of the genetic region *I35_RS03700–I35_RS03770* identified to be important in *B. cenocepacia* H111 for survival in *G. mellonella*. Open reading frames (ORFs) on forward and reverse strands are indicated by arrow directions, and gene locus tags are indicated. For comparison, the ortholog region in *B. cenocepacia* J2315 is shown.

Unique determinants for fitness in *G. mellonella* are genes involved in LPS biosynthesis, like a glucose-1-phosphate thymidylyltransferase (*BCAL3134, rmlA*), a sugar ABC transporter permease (*I35_RS03690*), and an ABC transporter ATP-binding protein (*BCAL3130, wzt*) (Table S2). Interestingly, most of these genes are detrimental in the EVPL model, i.e., inactivating them provides a fitness benefit. The LPS-related genes *waaL* (*BCAL2405*) and *wabRS* (*BCAL2406–BCAL2407*), involved in O-antigen ligation and core LPS assembly, were identified as being important for *G. mellonella* and detrimental for EVPL. However, the genes *wabPQ (BCAL2403–BCAL2404*) were important for the two host models. Examples of genes that were found to be unique for the wax moth larvae are a urease (*BCAL3103*, *BCAL3107, BCAL3108, ureDEF*), a protease (*BCAL1995–BCAL1996, clpXP*), and the twin-arginine translocation pathway (*BCAL0323–BCAL0325, tatABC*) (Fig. 3). Furthermore, we identified the gene cluster *I35_RS03700–I35_RS03770*, involved in LPS biosynthesis. An interesting aspect is that out of the 15 genes from this cluster, 13 were identified to be important in the *G. mellonella* model, while in the EVPL model, 11 out of those 13 were detrimental (Fig. 4). This large cluster constitutes a genomic island that is only present in a few *Bcc* strains of the species *B. cenocepacia*, *Burkholderia diffusa, Burkholderia stagnalis,* and *Burkholderia ubonensis*. The cluster is integrated between *wbiG* (*BCAL3121, a* putative nucleotide sugar epimerase/dehydratase) and *wzt* (*BCAL3130,* ABC transporter ATP-binding protein) (Fig. 4).

Among the unique fitness determinants for the EVPL model (Table S2) were genes required for tryptophan biosynthesis (*BCAM0990, BCAM0991, BCAM0993, trpABF; BCAL0396, BCAL0397, BCAL0399, trpCDE*). We also identified genes coding for an operon involved in NAD(+) biosynthesis (*BCAL2716–BCAL2718, nadBCA*), type 1 fimbrial proteins (*BCAL1677, BCAL1679*), a pyochelin receptor (*BCAM2224, fptA*), a major facilitator superfamily protein (*BCAM2221*) presumably involved in pyochelin transport (67), and a transcriptional regulator (*BCAM2231, pchR*) with homology to a pyochelin biosynthesis regulator (68) (Fig. 3). Furthermore, we found some chemotaxis-related proteins (*BCAM0821, BCAM0824*) involved in signal transduction (69) and gene *BCAL2981,* which codes for a hypothetical protein with homology to a tetratricopeptide repeat protein, which have been reported to be involved in protein-protein interactions, assembly of multiprotein complexes, and virulence (70–72).

### Validation of the Tn-seq results

**Fig 5:**
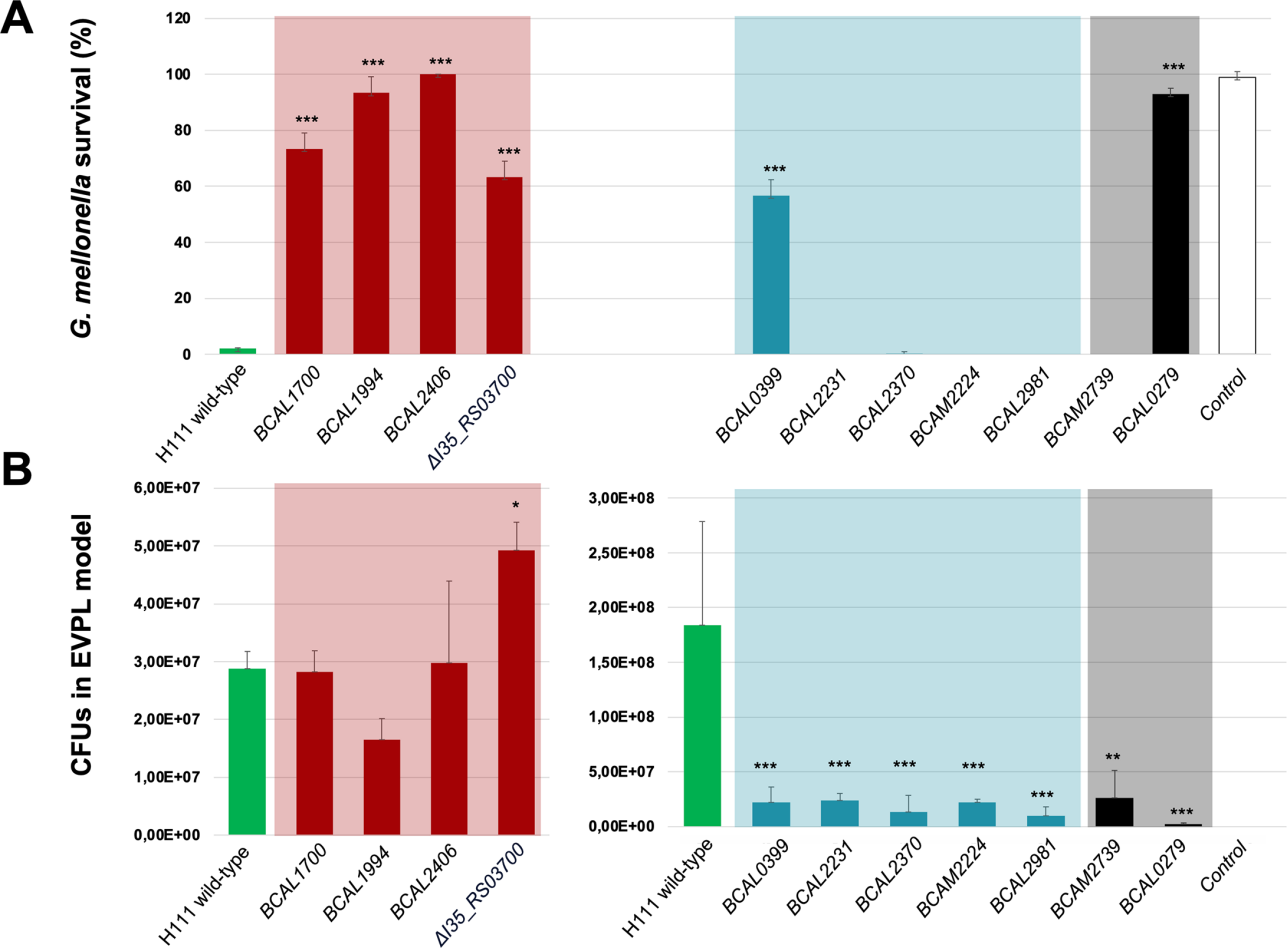
Validation of the Tn-seq host-associated fitness data in B*. cenocepacia* H111 using individual mutants. (A) Validation in *G. mellonella* represents the percentage of alive larvae after 72 h, (*n* = 3, 10 larvae each). PBS was used as a negative control. Data for *BCAL0279* were taken from Schwager et al. (19). (B) Validation in EVPL shows bacterial CFUs per lung as recovered after 48 h (*n* = 3, five pig lung pieces each). SCFM1 was used as a negative control. Significant difference to the *B. cenocepacia* H111 wild-type control is indicated by asterisks (one-way analysis of variance, Dunnett’s multiple comparison test): **, *P* < 0.01; ***, *P* < 0.001.

To evaluate the robustness of our screen and our Tn-seq host fitness data for *B. cenocepacia* H111, we constructed different mutants and assessed their fitness in the two models using the wild-type strain as control. For the genes uniquely important for survival in *G. mellonella*, we constructed mutants in *BCAL1700, BCAL1994, BCAL2403–BCAL2406,* and the *I35_RS03700–I35_RS03770* gene cluster (Fig. 5). *BCAL1700* (*orbA*) codes for an ornibactin receptor that is involved in iron acquisition via the siderophore ornibactin (69). *BCAL1994* (*lon*) codes for an ATP-dependent protease that shares 68% identity with the Lon protease of *Pseudomonas aeruginosa* (73). *BCAL2403–BCAL2406 (wabPQR, waaL*) code for genes involved in O-antigen ligation and core LPS assembly (74). Furthermore, we constructed a deletion mutant (Δ*I35_RS03700*) that lacks the complete *I35_RS03700–I35_RS03770* gene cluster (Fig. 4), potentially involved in LPS synthesis.

To validate genes uniquely important in the EVPL model, we constructed mutants in *BCAL2370, BCAL0399, BCAM2231, BCAM2224,* and *BCAL2981* (Fig. 5). *BCAL2370* codes for a hypothetical protein with homology to a glycosyl transferase. *BCAL0399* codes for anthranilate synthase component I (TrpE) involved in tryptophan synthesis. In *P. aeruginosa*, this pathway was shown to be important for virulence (75). *BCAM2231* codes for the family transcriptional regulator PchR*,* involved in pyochelin regulation (68); and *BCAM2224* codes for the pyochelin receptor *fptA. BCAL2981* codes for a hypothetical protein with homology to a tetratricopeptide repeat protein, previously shown to be involved in various functions such as protein folding and virulence (71, 72).

Regarding the genes important for both hosts, we chose the genes *BCAL0279* and *BCAM2739* for validation (Fig. 5). *BCAL0279* codes the shikimate kinase *aroK* and was previously shown to be involved in virulence in *G. mellonella* and other non-mammalian hosts (19). *BCAM2739* codes for a MoaA/NifB/PqqE family protein, with homology to the hopanoid biosynthesis associated radical SAM protein HpnH, which is essential for the production of hopanoids (76).

In agreement with our Tn-seq data, we observed that genes *BCAL1700, BCAL1994, BCAL2403–BCAL2406,* and *I35_RS03700–I35_RS03770* are important for virulence in *G. mellonella,* as the corresponding mutants were found to be attenuated. Expectedly, these mutants are not important for colonization of the EVPL model, as the CFUs recovered from the bronchiole tissue after 48 h were comparable to those obtained with the wild-type strain with the notable exception of mutant Δ*I35_RS03700* (Fig. 5, in red). In agreement with the finding that the *I35_RS03700–I35_RS03770* gene cluster showed a positive fitness benefit in our Tn-Seq analysis, we found that the deletion mutant showed significantly increased colonization of pig bronchiole tissue. The *BCAL1994* mutant colonized bronchiole tissue insignificantly less than the wild-type strain, in accordance with the slightly negative FC (−0.75) value observed in the Tn-seq screen.

We also validated genes *BCAL2370, BCAL0399, BCAM2231, BCAM2224, BCAL2981,* and *BCAM2739*, which were expected to be important for the colonization of the EVPL. The CFUs recovered from the bronchiole tissue after 48 h were for all mutants tested significantly lower than for the wild-type (Fig. 5, in turquoise), confirming our Tn-seq analysis. As we also predicted by the Tn-seq analysis, these genes were not important for fitness in *G. mellonella*, as 72 h post-infection, the numbers of dead larvae were virtually indistinguishable from those of the wild-type. Only mutant *BCAL0399* showed significantly attenuated killing. This could be due to the intermediate fold change value of this gene in *G. mellonella* (FC = −0.5), which is not strong enough to be considered an important gene in our lists (threshold FC ≤ −1) but obviously has some effect on fitness. Notably, gene *BCAL0398* is in the same operon as *BCAL0399,* which is among the core fitness determinants. Finally, we confirmed that the two mutants *BCAL0279* and *BCAM2739* are impaired in colonization in the EVPL model (Fig. 5, in gray), as predicted by Tn-Seq. Gene *BCAL0279* had previously been demonstrated to be important for survival in *G. mellonella* larvae (19). However, we could not confirm a role in virulence of *BCAM2739* in our *G. mellonella* infection model.

Our results demonstrate a high correlation between the Tn-seq high-throughput data and the individual mutant validation analysis. Collectively, they support the finding that some fitness determinants are model-specific while others are required for both hosts, highlighting the importance of using multiple models to unravel core host fitness genes.

### Phenotypic characterization of attenuated mutants

**Fig 6:**
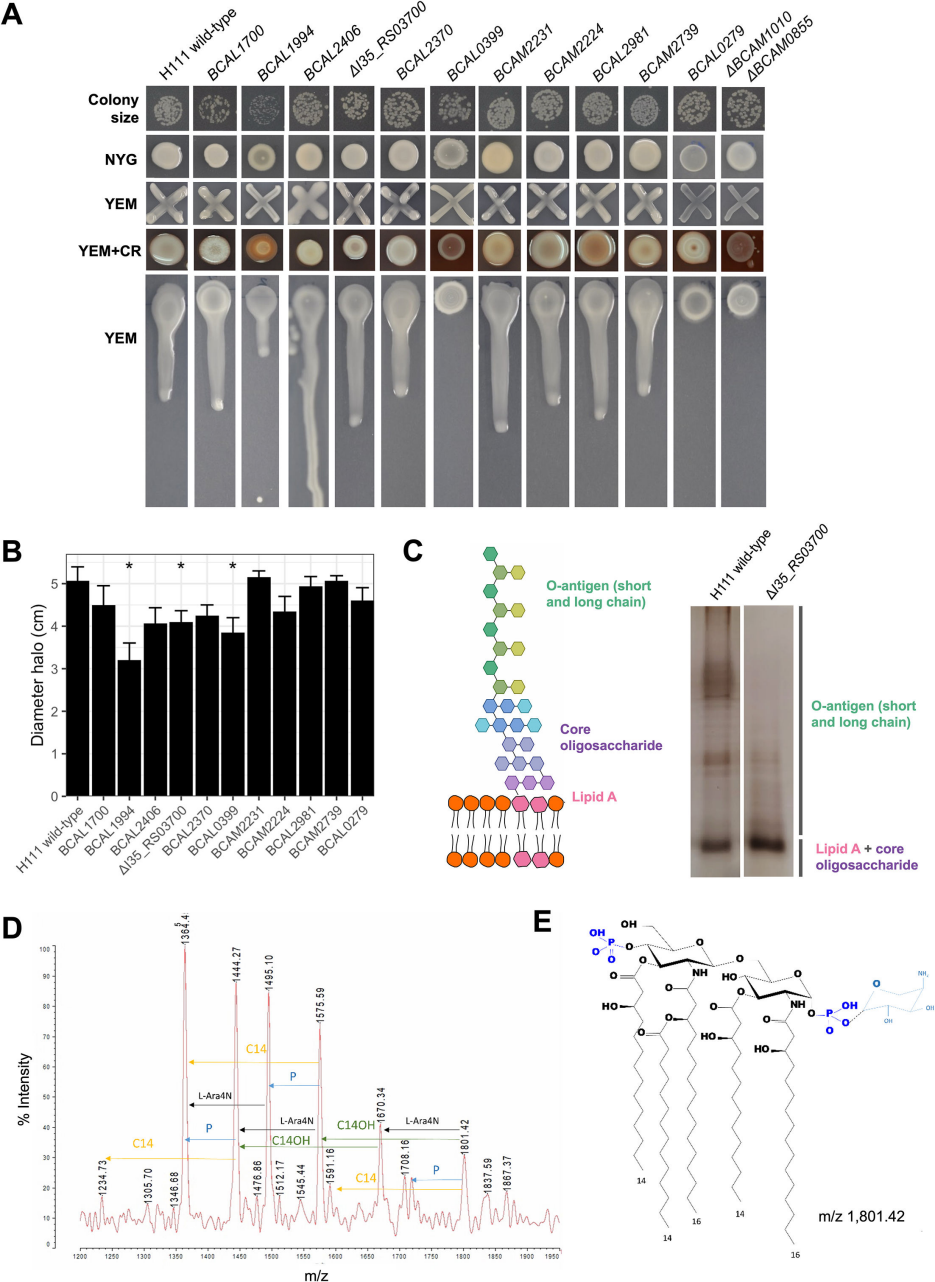
Phenotypic characterization of *B. cenocepacia* H111 and mutants. (A) Colony size, colony morphology (NYG medium), and EPS production (modified YEM medium ± CR). Mutant Δ*BCAM1010* Δ*BCAM0855*, defective in EPS production, was used as a control (59). (B) Swimming motility in LB media with 0.2% (wt/vol) agar. *t*-test, *P-value < 0.05. (C) LPS characterization of *B. cenocepacia* H111 wild-type and the Δ*I35_RS03700* mutant by polyacrylamide gel electrophoresis. (D) MALDI-TOF mass spectrum of the purified lipid A fraction of *B. cenocepacia* H111. (E) Structure of lipid A of *B. cenocepacia* H111.

To shed some light on the possible underlying mechanisms of the decreased fitness in the two models used, we tested the mutants for some phenotypic traits that have been reported to be important for host colonization. Mutants in genes *BCAL1994* and *BCAL2406* showed altered EPS production compared to the wild-type strain, albeit to a different degree (Fig. 6A); and most notably, no EPS production was observed in mutants *BCAL0399* and *BCAL0279*. These mutants also showed different colony morphology in NYG and/or YEM + CR medium. With regard to motility, mutants *BCAL1994*, Δ*I35_RS03700,* and *BCAL0399* were found to be significantly impaired in swimming relative to the wild-type strain, while mutants *BCAL2406* and *BCAL2370* showed a slight, although not significant, reduction (*P*-value = 0.056 and 0.07, respectively) (Fig. 6B; Fig. S1). Regarding sensitivity to the antimicrobial peptide PMB, which was used as a readout for genes involved in cell wall biogenesis, we observed that mutants *BCAL2403–BCAL2406, BCAL2981*, *BCAL1994,* and *BCAL2370* were much more sensitive to PMB (Fig. S2). The cell morphology and size of the mutants were indistinguishable from the wild-type when inspected by light microscopy (Fig. S3).

We also analyzed the LPS structure of mutant Δ*I35_RS03700* (Fig. 6). We observed by gel electrophoresis that the O-antigen of the LPS was affected in the mutant (Fig. 6C), which was shorter compared to the O-antigen of the wild-type. The composition of the lipid A moiety was further characterized by MALDI-TOF (Fig. 6D). The peak at m/z = 1,801.42 was likely due to a bis-phosphorylated glucosamine disaccharide backbone substituted with two 3-hydroxytetradecanoic acid [C14:0(3-OH)] residues in ester linkage and two 3-hydroxyhexadecanoic acid [C16:0(3-OH)] residues in amide linkage with a secondary tetradecanoic acid [C14:0] ester-linked fatty acid in position 2´ and one 4-amino-4-deoxy-arabinose residue attached to the phosphate group, which represents a penta-acylated lipid A (Fig. 6E). The spectrum of the mutant was found to be identical to the one of the wild-type strain (Fig. 6D), confirming that the only difference between wild-type and mutant strain is the O-antigen (Fig. 6C).

## DISCUSSION

In a previous study, we screened 5,500 *B. cenocepacia* H111 mini-Tn*5* insertion mutants for attenuated virulence in the *C. elegans* infection model (19). Although the use of *C. elegans* and other non-mammalian hosts allows screening of a large collection of mutants, this is a very laborious and time-consuming approach as an ordered mutant library has to be constructed and each mutant needs to be tested individually. With the advent of Tn-seq methodologies, it has become possible to perform genome-wide mutant screens in various infection models (39, 41–43, 77–81). Here, we employed Tn-seq to identify the fitness determinants in *B. cenocepacia* H111 required for survival in two animal models, *G. mellonella* larvae and a pig lung model (32, 34). Anatomically, the wax moth has a thick coating called cuticle, under which is found a thin epithelial layer. The inner cavity consists of the fat body as well as the hemolymph, the analog of the mammalian bloodstream. Regarding the EVPL, it consists of bronchiole lung tissue, lined with ciliated columnar epithelium, submerged in synthetic sputum medium. In this model, the anatomy, biochemistry, and physiology are comparable to that of the human lung. Work with other bacteria in these two different models suggests that *Galleria* mimics acute infection, while the EVPL model rather simulates a chronic infection. Additionally, an immune response is present in *G. mellonella* but not in the EVPL model (82). The innate immune system in *Galleria* shares many similarities with that of mammals. It includes both a cellular response with hemocytes—immune cells close to neutrophils—which are key actors responsible for cellular events, and a humoral response with soluble effector molecules (e.g., opsonins that target bacterial LPS and produce reactive oxygen species, lysozyme that targets bacterial peptidoglycan, and more than 20 different antimicrobial peptides) (82, 83).

Most of the genes that we identified in this study did not encode classic virulence factors but rather enzymes within metabolic pathways, including shikimate and amino acid biosynthesis. It is notable that these metabolic pathways were also identified in our previous *C. elegans* screen (19). Moreover, our Tn-seq data indicate that genes required for the biosynthesis of branched-chain amino acids, histidine, tryptophan, and biotin provide a fitness benefit for persistence in both host models. These results support previous findings that the ability to grow is critical to establish an infection, and that certain nutrients are limited in phylogenetically unrelated hosts (39, 84–93). Genes related to nutrient acquisition were also identified in a Tn-seq screen of *B. cenocepacia* performed in *C. elegans* (42) and of *Klebsiella pneumoniae* in *G. mellonella* (41). In agreement with our results, it has been shown that inactivation of the shikimate biosynthesis pathway in *Burkholderia pseudomallei*, the causative agent of melioidosis, renders this pathogen avirulent (94). It is therefore not surprising that depletion of intracellular tryptophan during invasion has been proposed as an effective antibacterial strategy in diverse hosts (95).

Previous work has identified three functions that are important for full pathogenicity of *B. cenocepacia* independent of the infection host: (i) LPS biosynthesis, (ii) QS, and (iii) siderophore production (12). Our data strongly support the importance of genes required for LPS biosynthesis for survival in both pathogenesis models, likely to withstand stresses such as pH and osmolarity as well as antimicrobial peptides produced by the hemolymph of *G. mellonella* and potentially by the pig lung tissue (96, 97). It has previously been shown in *B. cenocepacia* K56-2 that three loci are involved in the biosynthesis of O-antigen and protein glycosylation: the regions spanning *BCAL2401–BCAL2407 (wabOPQRS, waaL*), *BCAL0967 (waaF*) encoding a putative heptosyltransferase II, and *BCAL3109 (ureG)–BCAL3136* (*apaH*), which in addition contains genes involved in O-antigen biosynthesis (74). While the *BCAL2403–BCAL2407* gene cluster was found to provide a fitness benefit in the *G. mellonella* model, most of these genes were detrimental in the EVPL model. In agreement with previous findings (74), we show that a *BCAL2403–BCAL2406* mutant*,* which is defective in lipid A-core oligosaccharide biosynthesis, is not only attenuated in fitness in *Galleria* but also shows decreased resistance to PMB (Fig. 5A; Fig. S2). We also identified several genes involved in O-antigen biosynthesis (*BCAL3130, wzt; I35_RS03690; I35_RS03685, cpsB; BCAL3134, rmlA*) (Table S2) that appear to be important for survival in the wax moth larvae. Interestingly, except for *I35_RS03685,* these genes had positive FC values in the EVPL model, indicating that their inactivation is beneficial for the colonization of pig lung tissue. This is in line with previous work showing that loss of O-antigen production in *B. cenocepacia* K56-2 increased bacterial adherence to A549 human lung epithelial cells, likely because the O-antigen masks bacterial surface molecules that can interact with epithelial cell receptors (74). These results show that the effect of some fitness determinants is strongly dependent on the host type used, and that, while some genes provide a fitness benefit in one infection model, they may be disadvantageous in another one.

With regard to QS, we only found indirect evidence that it may play a role in the persistence of *B. cenocepacia* H111 in the EVPL model, as we identified two QS-regulated genes in our Tn-seq screen, *BCAL1677*, which codes for a type 1 fimbrial protein with homology to FimA, a major subunit of type 1 fimbriae of *E. coli*, and *BCAL1679*, one of three downstream genes related to a putative fimbrial chaperone pathway (98). The reason that our analysis failed to identify QS-regulated functions known to be important for virulence is that many of these genes encode public goods, which cannot be identified by Tn-seq with the host models used, which is a clear limitation of the Tn-seq approach (39).

Regarding the involvement of iron acquisition genes, it is a well-documented bacterial strategy for the colonization of the CF lung (99–101), being the mucus of chronically infected patients rich in iron. *B. cenocepacia* H111 relies on two different siderophores, ornibactin and pyochelin (99, 101, 102). Pyochelin is less widespread than ornibactin among *Bcc* strains isolated from CF patients (103). While both siderophores contribute to the virulence of strain H111, the effect of ornibactin was shown to be more prominent given its higher iron binding affinity (101, 104). Our Tn-seq analysis identified several genes (*orbSEIJAL* and *pvdA*) required for biosynthesis, uptake, and utilization of ornibactin in the *G. mellonella* model, confirming the importance of this siderophore for virulence in this infection model. Notably, these genes were identified even though siderophores are considered public good and thus should not be detectable by Tn-seq (see above). However, it was recently shown that cheating of such a common good is prevented in H111 due to the ornibactin receptor and synthesis genes being co‐expressed in the same operon and, as a consequence, receptor availability being coupled with siderophore production (105). This explains why we find these genes in our screening. The importance of ornibactin for pathogenicity was further confirmed by demonstrating that an *orbA* mutant is attenuated in the *G. mellonella* model (Fig. 5). Interestingly, ornibactin was found to be unimportant for the colonization of pig lung tissue. However, our data suggest that *BCAM2221*, *BCAM2224,* and *BCAM2231*, related to pyochelin transport, reception (*fptA*), and regulation (*pchR*), are important for lung colonization. As expected, we could not identify genes involved in pyochelin synthesis (see common goods explanation above), but the finding that mutants with mutations in genes required for the regulation and uptake of pyochelin are depleted from the library suggests that pyochelin is important for fitness in pig lung tissue, although not for acute virulence in *G. mellonella*. In agreement with this, we could confirm that *BCAM2224* (*fptA*) and *BCAM2231* (*pchR*) mutants are impaired in pig lung tissue colonization while they were unaffected in virulence in *G. mellonella* 72 h after infection (Fig. 5). Collectively, our results may explain why *B. cenocepacia* H111 can produce two different types of siderophores, which provide differential benefits depending on the host environment.

In conclusion, our Tn-seq screening has identified both already-known and novel genes in *B. cenocepacia* H111 that are important for fitness the pig lung and *G. mellonella* infection models. While there is a substantial overlap of the fitness determinants that are important for the two host types, we also identified genes only required in one of the hosts. This pinpoints the importance of using different models to assess virulence and identify infection factors. Most intriguingly, we show that a rare genomic island (*I35_RS03700*–*I35_RS03770*), which is only found in a few Bcc strains and codes for enzymes involved in LPS O-antigen biosynthesis, is important in for the colonization of the *G. mellonella* and detrimental for the EVPL model. This strongly underpins the idea that LPS is a key factor for virulence, as has been demonstrated in numerous studies (12, 106–109). It is noteworthy that most *in vivo* and *ex vivo* models used nowadays have been optimized for bacteria different from *Burkholderia* (mainly *P. aeruginosa*). Therefore, it is not clear what aspects of the real host-pathogen relationship they capture for the pathogen *B. cenocepacia*, and we hope that this work will help toward understanding them. Finally, our study also supports evidence that nutrient availability in the host during colonization is limited and thus the *de novo* biosynthesis of certain amino acids such as tryptophan is a general requirement for pathogenesis (39). Enzymes involved in all the above pathways could be valuable targets for the development of novel antibacterial drugs.

## Data Availability

Raw FASTQ files generated from the Illumina MiSeq platform are publicly available from the NCBI short reads archive (SRA) and can be found using the following link: https://www.ncbi.nlm.nih.gov/sra/PRJNA1220324. Raw FASTQ MiSeq data corresponding to the control in LB (47) is available at https://www.ncbi.nlm.nih.gov/bioproject/PRJNA397863 under accession SRX3085715.

## SUPPLEMENTAL MATERIAL

Supplemental figures msystems.01354-24-s0001.pdf Fig. S1 to S3.

Table S1 msystems.01354-24-s0002.xlsx Bacterial strains, oligonucleotides, and plasmids used in this study.

Table S2 msystems.01354-24-s0003.xlsx *Burkholderia cenocepacia* H111 fitness genes and shared and unique genes in the *Galleria mellonella* and EVPL models.

ASM does not own the copyrights to Supplemental Material that may be linked to, or accessed through, an article. The authors have granted ASM a non-exclusive, world-wide license to publish the Supplemental Material files. Please contact the corresponding author directly for reuse.

## References

[1] De Volder AL, Teves S, Isasmendi A, Pinheiro JL, Ibarra L, Breglia N, Herrera T, Vazquez M, Hernandez C, Degrossi J. 2021. Distribution of Burkholderia cepacia complex species isolated from industrial processes and contaminated products in Argentina. Int Microbiol 24:157–167. doi:10.1007/s10123-020-00151-z 33184776

[2] Eberl L, Vandamme P. 2016. Members of the genus Burkholderia: good and bad guys. F1000Res 5:F1000 Faculty Rev-1007. doi:10.12688/f1000research.8221.1 PMC4882756 27303639

[3] Vanlaere E, Baldwin A, Gevers D, Henry D, Brandt E, LiPuma JJ. 2009. Taxon K, a complex within the Burkholderia cepacia complex, comprises at least two novel species. Int J Syst Evol Microbiol 59:102–111. doi:10.1099/ijs.0.001123-0 19126732

[4] Suárez-Moreno ZR, Caballero-Mellado J, Coutinho BG, Mendonça-Previato L, James EK, Venturi V. 2012. Common features of environmental and potentially beneficial plant-associated Burkholderia*.* Microb Ecol 63:249–266. doi:10.1007/s00248-011-9929-1 21850446

[5] Mahenthiralingam E, Urban TA, Goldberg JB. 2005. The multifarious, multireplicon Burkholderia cepacia complex. Nat Rev Microbiol 3:144–156. doi:10.1038/nrmicro1085 15643431

[6] Vial L, Chapalain A, Groleau MC, Déziel E. 2011. The various lifestyles of the Burkholderia cepacia complex species: a tribute to adaptation. Environ Microbiol 13:1–12. doi:10.1111/j.1462-2920.2010.02343.x 20880095

[7] Srinivasan LCS, Arora M, Sahai CK. 2016. Report on the newly emerging nosocomial Burkholderia cepacia in a tertiary hospital. Med J Armed Forces India 72:S50–S53. doi:10.1016/j.mjafi.2016.03.003 28050070 PMC5192204

[8] Viderman D, Khudaibergenova M, Kemaikin V, Zhumadilov A, Poddighe D. 2020. Outbreak of catheter-related Burkholderia cepacia sepsis acquired from contaminated ultrasonography gel: the importance of strengthening hospital infection control measures in low resourced settings. Le Infez Med 28:551–557. 33257630

[9] Kandemir O, Erbey F, Asarcıklı F, Sözmen BO, Özen MA, Aktürk H. 2022. Catheter-related bloodstream infection caused by Burkholderia cepacia in a pediatric oncology patient. Germs 12:316–317. doi:10.18683/germs.2022.1335 36504612 PMC9719382

[10] Cheng L-K, Chau S-Y, Chan W-S, Chen J-K, Wong B-C, Fung K-C. 2024. An outbreak of Burkholderia cepacia complex exit site infection among peritoneal dialysis patients caused by contaminated spray dressing. Infect Prev Pract 6:100359. doi:10.1016/j.infpip.2024.100359 38559368 PMC10981104

[11] Saeed M, Rasheed F, Rasool MH, Hayat S, Khurshid M. 2024. Carbapenem‐resistant Burkholderia cepacia complex isolates carrying blaNDM−1 and blaNDM−5 in ventilator‐associated pneumonia patients and contaminated ventilator tubing. Transbound Emerg Dis. doi:10.1155/2024/3352135 PMC12016991 40303040

[12] Uehlinger S, Schwager S, Bernier SP, Riedel K, Nguyen DT, Sokol PA, Eberl L. 2009. Identification of specific and universal virulence factors in Burkholderia cenocepacia strains by using multiple infection hosts. Infect Immun 77:4102–4110. doi:10.1128/IAI.00398-09 19528212 PMC2738042

[13] Huber B, Feldmann F, Köthe M, Vandamme P, Wopperer J, Riedel K, Eberl L. 2004. Identification of a novel virulence factor in Burkholderia cenocepacia H111 required for efficient slow killing of Caenorhabditis elegans. Infect Immun 72:7220–7230. doi:10.1128/IAI.72.12.7220-7230.2004 15557647 PMC529107

[14] Drevinek P, Mahenthiralingam E. 2010. Burkholderia cenocepacia in cystic fibrosis: epidemiology and molecular mechanisms of virulence. Clin Microbiol Infect 16:821–830. doi:10.1111/j.1469-0691.2010.03237.x 20880411

[15] Loutet SA, Valvano MA. 2010. A decade of Burkholderia cenocepacia virulence determinant research. Infect Immun 78:4088–4100. doi:10.1128/IAI.00212-10 20643851 PMC2950345

[16] Geisenberger O, Givskov M, Riedel K, Høiby N, Tümmler B, Eberl L. 2000. Production of N-acyl-L-homoserine lactones by P. aeruginosa isolates from chronic lung infections associated with cystic fibrosis. FEMS Microbiol Lett 184:273–278. doi:10.1111/j.1574-6968.2000.tb09026.x 10713433

[17] Gotschlich A, Huber B, Geisenberger O, Tögl A, Steidle A, Riedel K, Hill P, Tümmler B, Vandamme P, Middleton B, Camara M, Williams P, Hardman A, Eberl L. 2001. Synthesis of multiple N-acylhomoserine lactones is wide-spread among the members of the Burkholderia cepacia complex. Syst Appl Microbiol 24:1–14. doi:10.1078/0723-2020-00013 11403388

[18] Pessi G, Braunwalder R, Grunau A, Omasits U, Ahrens CH, Eberl L. 2013. Response of Burkholderia cenocepacia H111 to micro-oxia. PLoS One 8:e72939. doi:10.1371/journal.pone.0072939 24023794 PMC3759415

[19] Schwager S, Agnoli K, Köthe M, Feldmann F, Givskov M, Carlier A, Eberl L. 2013. Identification of Burkholderia cenocepacia strain H111 virulence factors using nonmammalian infection hosts. Infect Immun 81:143–153. doi:10.1128/IAI.00768-12 23090963 PMC3536147

[20] Köthe M, Antl M, Huber B, Stoecker K, Ebrecht D, Steinmetz I, Eberl L. 2003. Killing of caenorhabditis elegans by Burkholderia cepacia is controlled by the cep quorum-sensing system. Cell Microbiol 5:343–351. doi:10.1046/j.1462-5822.2003.00280.x 12713492

[21] Huber B, Riedel K, Köthe M, Givskov M, Molin S, Eberl L. 2002. Genetic analysis of functions involved in the late stages of biofilm development in Burkholderia cepacia H111. Mol Microbiol 46:411–426. doi:10.1046/j.1365-2958.2002.03182.x 12406218

[22] Carlier A, Agnoli K, Pessi G, Suppiger A, Jenul C, Schmid N, Tümmler B, Pinto-Carbo M, Eberl L. 2014. Genome sequence of Burkholderia cenocepacia H111, a cystic fibrosis airway isolate. Genome Announc 2:e00298-14. doi:10.1128/genomeA.00298-14 24723723 PMC3983312

[23] Ensinck M, Mottais A, Detry C, Leal T, Carlon MS. 2021. On the corner of models and cure: gene editing in cystic fibrosis. Front Pharmacol 12:662110. doi:10.3389/fphar.2021.662110 33986686 PMC8111007

[24] Roda J, Pinto-Silva C, Silva IAI, Maia C, Almeida S, Ferreira R, Oliveira G. 2022. New drugs in cystic fibrosis: what has changed in the last decade? Ther Adv Chronic Dis 13:20406223221098136. doi:10.1177/20406223221098136 35620188 PMC9128052

[25] Chua I-L, Blyth C, Porter M, Minney-Smith C, Merritt A, Brennan B, Farrar C, Pelumbo L, Engelbrecht D, Nicolaou L. 2024. The brief case Burkholderia cepacia infection in the neonatal unit: a cautionary tale of contaminated EEG skin preparation. Int J infect control 20:3–5. doi:10.3396/ijic.v20.23528

[26] Cutuli MA, Petronio Petronio G, Vergalito F, Magnifico I, Pietrangelo L, Venditti N, Di Marco R. 2019. Galleria mellonella as a consolidated in vivo model hosts: new developments in antibacterial strategies and novel drug testing. Virulence 10:527–541. doi:10.1080/21505594.2019.1621649 31142220 PMC6550544

[27] Castonguay-Vanier J, Vial L, Tremblay J, Déziel E. 2010. Drosophila melanogaster as a model host for the Burkholderia cepacia complex. PLoS One 5:e11467. doi:10.1371/journal.pone.0011467 20635002 PMC2902503

[28] Deng Y, Boon C, Eberl L, Zhang LH. 2009. Differential modulation of Burkholderia cenocepacia virulence and energy metabolism by the quorum-sensing signal BDSF and its synthase. J Bacteriol 191:7270–7278. doi:10.1128/JB.00681-09 19801414 PMC2786553

[29] Seed KD, Dennis JJ. 2008. Development of galleria mellonella as an alternative infection model for the Burkholderia cepacia complex. Infect Immun 76:1267–1275. doi:10.1128/IAI.01249-07 18195031 PMC2258804

[30] Vergunst AC, Meijer AH, Renshaw SA, O’Callaghan D. 2010. Burkholderia cenocepacia creates an intramacrophage replication niche in zebrafish embryos, followed by bacterial dissemination and establishment of systemic infection. Infect Immun 78:1495–1508. doi:10.1128/IAI.00743-09 20086083 PMC2849400

[31] Trevijano-Contador N, Zaragoza O. 2018. Immune response of Galleria mellonella against human fungal pathogens. J Fungi (Basel) 5:1–13. doi:10.3390/jof5010003 30587801 PMC6463112

[32] Harrison F, Muruli A, Higgins S, Diggle SP. 2014. Development of an ex vivo porcine lung model for studying growth virulence, and signaling of Pseudomonas aeruginosa. Infect Immun 82:3312–3323. doi:10.1128/IAI.01554-14 24866798 PMC4136229

[33] Harrington NE, Sweeney E, Harrison F. 2020. Building a better biofilm - formation of in vivo-like biofilm structures by Pseudomonas aeruginosa in a porcine model of cystic fibrosis lung infection. Biofilm 2:100024. doi:10.1016/j.bioflm.2020.100024 33381751 PMC7762787

[34] Harrison F, Diggle SP. 2016. An ex vivo lung model to study bronchioles infected with Pseudomonas aeruginosa biofilms. Microbiology (Reading) 162:1755–1760. doi:10.1099/mic.0.000352 27520088

[35] Goodman AL, McNulty NP, Zhao Y, Leip D, Mitra RD, Lozupone CA, Knight R, Gordon JI. 2009. Identifying genetic determinants needed to establish a human gut symbiont in its habitat. Cell Host Microbe 6:279–289. doi:10.1016/j.chom.2009.08.003 19748469 PMC2895552

[36] van Opijnen T, Bodi KL, Camilli A. 2009. Tn-seq: high-throughput parallel sequencing for fitness and genetic interaction studies in microorganisms. Nat Methods 6:767–772. doi:10.1038/nmeth.1377 19767758 PMC2957483

[37] Langridge GC, Phan M-D, Turner DJ, Perkins TT, Parts L, Haase J, Charles I, Maskell DJ, Peters SE, Dougan G, Wain J, Parkhill J, Turner AK. 2009. Simultaneous assay of every Salmonella Typhi gene using one million transposon mutants. Genome Res 19:2308–2316. doi:10.1101/gr.097097.109 19826075 PMC2792183

[38] Gawronski JD, Wong SMS, Giannoukos G, Ward DV, Akerley BJ. 2009. Tracking insertion mutants within libraries by deep sequencing and a genome-wide screen for Haemophilus genes required in the lung. Proc Natl Acad Sci U S A 106:16422–16427. doi:10.1073/pnas.0906627106 19805314 PMC2752563

[39] Torres M, Paszti S, Eberl L. 2024. Shedding light on bacteria-host interactions with the aid of TnSeq approaches. mBio 15:e00390-24. doi:10.1128/mbio.00390-24 38722161 PMC11237515

[40] Fan J, Zhao L, Hu Q, Li S, Li H, Zhang Q, Zou G, Zhang L, Li L, Huang Q, Zhou R. 2022. Screening for virulence-related genes via a transposon mutant library of streptococcus suis serotype 2 using a galleria mellonella larvae infection model. Microorganisms 10:868. doi:10.3390/microorganisms10050868 35630313 PMC9143085

[41] Bruchmann S, Feltwell T, Parkhill J, Short FL. 2021. Identifying virulence determinants of multidrug-resistant klebsiella pneumoniae in galleria mellonella. Pathog Dis 79:1–15. doi:10.1093/femspd/ftab009 PMC7981267 33512418

[42] Wong Y-C, Abd El Ghany M, Ghazzali RNM, Yap S-J, Hoh C-C, Pain A, Nathan S. 2018. Genetic determinants associated with in Vivo survival of Burkholderia cenocepacia in the caenorhabditis elegans model. Front Microbiol 9:1–14. doi:10.3389/fmicb.2018.01118 29896180 PMC5987112

[43] Torres M, Jiquel A, Jeanne E, Naquin D, Dessaux Y, Faure D. 2022. Agrobacterium tumefaciens fitness genes involved in the colonization of plant tumors and roots. New Phytol 233:905–918. doi:10.1111/nph.17810 34655498

[44] Rosconi F, Rudmann E, Li J, Surujon D, Anthony J, Frank M, Jones DS, Rock C, Rosch JW, Johnston CD, van Opijnen T. 2022. A bacterial pan-genome makes gene essentiality strain-dependent and evolvable. Nat Microbiol 7:1580–1592. doi:10.1038/s41564-022-01208-7 36097170 PMC9519441

[45] Jeffrey H. M. 1972. Experiments in molecular genetics. In Cold Spring Harbor, N. Y

[46] Neve RL, Carrillo BD, Phelan VV. 2021. Impact of artificial sputum medium formulation on Pseudomonas aeruginosa secondary metabolite production. J Bacteriol 203:e00250-21. doi:10.1128/JB.00250-21 34398662 PMC8508215

[47] Higgins S, Sanchez-Contreras M, Gualdi S, Pinto-Carbó M, Carlier A, Eberl L. 2017. The essential genome of Burkholderia cenocepacia H111. J Bacteriol 199:e00260-17. doi:10.1128/JB.00260-17 28847919 PMC5648868

[48] Paszti S, Vitale A, Liu Y, Braunwalder R, Kalawong R, Biner O, Pessi G, Eberl L. 2022. Identification of key factors for anoxic survival of B. cenocepacia H111. Int J Mol Sci 23:4560. doi:10.3390/ijms23094560 35562951 PMC9104464

[49] Gallagher LA, Shendure J, Manoil C. 2011. Resistance functions in Pseudomonas aeruginosa using Tn-seq 2:1–8. doi:10.1128/mBio.00315-10 PMC3023915 21253457

[50] Gallagher LA, Ramage E, Patrapuvich R, Weiss E, Brittnacher M, Manoil C. 2013. Sequence-defined transposon mutant library of Burkholderia thailandensis. mBio 4:e00604-13. doi:10.1128/mBio.00604-13 PMC3870259 24194535

[51] Vitale A, Paszti S, Takahashi K, Toyofuku M, Pessi G, Eberl L. 2020. Mapping of the denitrification pathway in Burkholderia thailandensis by genome-wide mutant profiling. J Bacteriol 202:e00304-20. doi:10.1128/JB.00304-20 32900830 PMC7648151

[52] Solaimanpour S, Sarmiento F, Mrázek J. 2015. Tn-seq explorer: a tool for analysis of high-throughput sequencing data of transposon mutant libraries. PLoS One 10:e0126070. doi:10.1371/journal.pone.0126070 25938432 PMC4418687

[53] Holden MTG, Seth-Smith HMB, Crossman LC, Sebaihia M, Bentley SD, Cerdeño-Tárraga AM, Thomson NR, Bason N, Quail MA, Sharp S, et al.. 2009. The genome of Burkholderia cenocepacia J2315, an epidemic pathogen of cystic fibrosis patients. J Bacteriol 191:261–277. doi:10.1128/JB.01230-08 18931103 PMC2612433

[54] Huerta-Cepas J, Szklarczyk D, Heller D, Hernández-Plaza A, Forslund SK, Cook H, Mende DR, Letunic I, Rattei T, Jensen LJ, von Mering C, Bork P. 2019. eggNOG 5.0: A hierarchical, functionally and phylogenetically annotated orthology resource based on 5090 organisms and 2502 viruses. Nucleic Acids Res 47:D309–D314. doi:10.1093/nar/gky1085 30418610 PMC6324079

[55] Lardi M, Liu Y, Purtschert G, Bolzan de Campos S, Pessi G. 2017. Transcriptome analysis of Paraburkholderia phymatum under nitrogen starvation and during symbiosis with phaseolus vulgaris. Genes (Basel) 8:389. doi:10.3390/genes8120389 29244728 PMC5748707

[56] Flannagan RS, Linn T, Valvano MA. 2008. A system for the construction of targeted unmarked gene deletions in the genus Burkholderia. Environ Microbiol 10:1652–1660. doi:10.1111/j.1462-2920.2008.01576.x 18341581

[57] Deng Y, Schmid N, Wang C, Wang J, Pessi G, Wu D, Lee J, Aguilar C, Ahrens CH, Chang C, Song H, Eberl L, Zhang L-H. 2012. Cis-2-dodecenoic acid receptor RpfR links quorum-sensing signal perception with regulation of virulence through cyclic dimeric guanosine monophosphate turnover. Proc Natl Acad Sci USA 109:15479–15484. doi:10.1073/pnas.1205037109 22949660 PMC3458384

[58] Richau JA, Leitão JH, Correia M, Lito L, Salgado MJ, Barreto C, Cescutti P, Sá-Correia I. 2000. Molecular typing and exopolysaccharide biosynthesis of Burkholderia cepacia isolates from a Portuguese cystic fibrosis center. J Clin Microbiol 38:1651–1655. doi:10.1128/JCM.38.4.1651-1655.2000 10747161 PMC86514

[59] Steiner E, Shilling RE, Richter AM, Schmid N, Fazli M, Kaever V, Jenal U, Tolker-Nielsen T, Eberl L. 2022. The BDSF quorum sensing receptor RpfR regulates Bep exopolysaccharide synthesis in Burkholderia cenocepacia via interaction with the transcriptional regulator BerB. NPJ Biofilms Microbiomes 8:93. doi:10.1038/s41522-022-00356-2 36418316 PMC9684580

[60] Westphal O. 1965. Bacterial lipopolysaccharides: extraction with phenol-water and further applications of the procedure. Methods Carbohydr Chem 5:83. doi:10.1007/978-90-481-9078-2_2

[61] Laemmli UK. 1970. Cleavage of structural proteins during the assembly of the head of bacteriophage T4. Nature 227:680–685. doi:10.1038/227680a0 5432063

[62] Tsai C-M, Frasch CE. 1982. A sensitive silver stain for detecting lipopolysaccharides in polyacrylamide gels. Anal Biochem 119:115–119. doi:10.1016/0003-2697(82)90673-X 6176137

[63] Chafchaouni‐Moussaoui I, Novikov A, Bhrada F, Perry MB, Filali‐Maltouf A, Caroff M. 2011. A new rapid and micro‐scale hydrolysis, using triethylamine citrate, for lipopolysaccharide characterization by mass spectrometry. Rapid Comm Mass Spectrometry 25:2043–2048. doi:10.1002/rcm.5084 21698687

[64] Cain AK, Barquist L, Goodman AL, Paulsen IT, Parkhill J, van Opijnen T. 2020. A decade of advances in transposon-insertion sequencing. Nat Rev Genet 21:526–540. doi:10.1038/s41576-020-0244-x 32533119 PMC7291929

[65] Oppy CC, Jebeli L, Kuba M, Oates CV, Strugnell R, Edgington-Mitchell LE, Valvano MA, Hartland EL, Newton HJ, Scott NE. 2019. Loss of O-linked protein glycosylation in Burkholderia cenocepacia impairs biofilm formation and siderophore activity and alters transcriptional regulators. mSphere 4:e00660-19. doi:10.1128/mSphere.00660-19 31722994 PMC6854043

[66] Fathy Mohamed Y, Scott NE, Molinaro A, Creuzenet C, Ortega X, Lertmemongkolchai G, Tunney MM, Green H, Jones AM, DeShazer D, Currie BJ, Foster LJ, Ingram R, De Castro C, Valvano MA. 2019. A general protein O-glycosylation machinery conserved in Burkholderia species improves bacterial fitness and elicits glycan immunogenicity in humans. J Biol Chem 294:13248–13268. doi:10.1074/jbc.RA119.009671 31350337 PMC6737235

[67] Thomas MS. 2007. Iron acquisition mechanisms of the Burkholderia cepacia complex. Biometals 20:431–452. doi:10.1007/s10534-006-9065-4 17295049

[68] Heinrichs DE, Poole K. 1996. PchR, a regulator of ferripyochelin receptor gene (fptA) expression in Pseudomonas aeruginosa, functions both as an activator and as a repressor. J Bacteriol 178:2586–2592. doi:10.1128/jb.178.9.2586-2592.1996 8626326 PMC177983

[69] Winsor GL, Khaira B, Van Rossum T, Lo R, Whiteside MD, Brinkman FSL. 2008. The Burkholderia genome database: facilitating flexible queries and comparative analyses. Bioinformatics 24:2803–2804. doi:10.1093/bioinformatics/btn524 18842600 PMC2639269

[70] Blatch GL, Lässle M. 1999. The tetratricopeptide repeat: a structural motif mediating protein-protein interactions. Bioessays 21:932–939. doi:10.1002/(SICI)1521-1878(199911)21:11<932::AID-BIES5>3.0.CO;2-N 10517866

[71] D’Andrea LD, Regan L. 2003. TPR proteins: the versatile helix. Trends Biochem Sci 28:655–662. doi:10.1016/j.tibs.2003.10.007 14659697

[72] Cerveny L, Straskova A, Dankova V, Hartlova A, Ceckova M, Staud F, Stulik J. 2013. Tetratricopeptide repeat motifs in the world of bacterial pathogens: role in virulence mechanisms. Infect Immun 81:629–635. doi:10.1128/IAI.01035-12 23264049 PMC3584863

[73] Agarwala R, Barrett T, Beck J, Benson DA, Bollin C, Bolton E, Bourexis D, Brister JR, Bryant SH, Canese K. 2018. Database resources of the national center for biotechnology information. Nucleic Acids Res 46:D8–D13. doi:10.1093/nar/gkx1095 29140470 PMC5753372

[74] Ortega X, Silipo A, Saldías MS, Bates CC, Molinaro A, Valvano MA. 2009. Biosynthesis and structure of the Burkholderia cenocepacia K56-2 lipopolysaccharide core oligosaccharide: truncation of the core oligosaccharide leads to increased bbinding and sensitivity to polymyxin B. J Biol Chem 284:21738–21751. doi:10.1074/jbc.M109.008532 19525227 PMC2755896

[75] Palmer GC, Jorth PA, Whiteley M. 2013. The role of two Pseudomonas aeruginosa anthranilate synthases in tryptophan and quorum signal production. Microbiology (Reading) 159:959–969. doi:10.1099/mic.0.063065-0 23449919 PMC3709558

[76] Schmerk CL, Welander PV, Hamad MA, Bain KL, Bernards MA, Summons RE, Valvano MA. 2015. Elucidation of the Burkholderia cenocepacia hopanoid biosynthesis pathway uncovers functions for conserved proteins in hopanoid-producing bacteria. Environ Microbiol 17:735–750. doi:10.1111/1462-2920.12509 24888970

[77] Ibberson CB, Stacy A, Fleming D, Dees JL, Rumbaugh K, Gilmore MS, Whiteley M. 2017. Co-infecting microorganisms dramatically alter pathogen gene essentiality during polymicrobial infection. Nat Microbiol 2:17079. doi:10.1038/nmicrobiol.2017.79 28555625 PMC5774221

[78] de Vries SP, Gupta S, Baig A, Wright E, Wedley A, Jensen AN, Lora LL, Humphrey S, Skovgård H, Macleod K, Pont E, Wolanska DP, L’Heureux J, Mobegi FM, Smith DGE, Everest P, Zomer A, Williams N, Wigley P, Humphrey T, Maskell DJ, Grant AJ. 2017. Genome-wide fitness analyses of the foodborne pathogen campylobacter jejuni in in vitro and in vivo models. Sci Rep 7:1251. doi:10.1038/s41598-017-01133-4 28455506 PMC5430854

[79] Fu Y, Waldor MK, Mekalanos JJ. 2013. Tn-Seq analysis of vibrio cholerae intestinal colonization reveals a role for T6SS-mediated antibacterial activity in the host. Cell Host Microbe 14:652–663. doi:10.1016/j.chom.2013.11.001 24331463 PMC3951154

[80] Gonyar LA, Gelbach PE, McDuffie DG, Koeppel AF, Chen Q, Lee G, Temple LM, Stibitz S, Hewlett EL, Papin JA, Damron FH, Eby JC. 2019. In vivo gene essentiality and metabolism in Bordetella pertussis. mSphere 4:1359–1363. doi:10.1128/mSphere.00694-18 PMC6531889 31118307

[81] Wheatley RM, Ford BL, Li L, Aroney STN, Knights HE, Ledermann R, East AK, Ramachandran VK, Poole PS. 2020. Lifestyle adaptations of Rhizobium from rhizosphere to symbiosis. Proc Natl Acad Sci U S A 117:23823–23834. doi:10.1073/pnas.2009094117 32900931 PMC7519234

[82] O’Toole GA, Crabbé A, Kümmerli R, LiPuma JJ, Bomberger JM, Davies JC, Limoli D, Phelan VV, Bliska JB, DePas WH, et al.. 2021. Model systems to study the chronic, polymicrobial infections in cystic fibrosis: current approaches and exploring future directions. mBio 12:e01763-21. doi:10.1128/mBio.01763-21 34544277 PMC8546538

[83] Ménard G, Rouillon A, Cattoir V, Donnio PY. 2021. Galleria mellonella as a suitable model of bacterial infection: past, present and future. Front Cell Infect Microbiol 11:782733. doi:10.3389/fcimb.2021.782733 35004350 PMC8727906

[84] Brubaker RR. 1970. Interconversion of purine mononucleotides in Pasteurella pestis. Infect Immun 1:446–454. doi:10.1128/iai.1.5.446-454.1970 16557756 PMC415922

[85] Alcantara RB, Read RDA, Valderas MW, Brown TD, Roop RM 2nd. 2004. Intact purine biosynthesis pathways are required for wild-type virulence of Brucella abortus 2308 in the BALB/c mouse model. Infect Immun 72:4911–4917. doi:10.1128/IAI.72.8.4911-4917.2004 15271960 PMC470684

[86] Crawford RM, Van De Verg L, Yuan L, Hadfield TL, Warren RL, Drazek ES, Houng HH, Hammack C, Sasala K, Polsinelli T, Thompson J, Hoover DL. 1996. Deletion of pure attenuates brucella melitensis infection in mice. Infect Immun 64:2188–2192. doi:10.1128/iai.64.6.2188-2192.1996 8675325 PMC174054

[87] Goncheva Mariya I, Flannagan RS, Sterling BE, Laakso HA, Friedrich NC, Kaiser JC, Watson DW, Wilson CH, Sheldon JR, McGavin MJ, Kiser PK, Heinrichs DE. 2019. Stress-induced inactivation of the Staphylococcus aureus purine biosynthesis repressor leads to hypervirulence. Nat Commun 10:775. doi:10.1038/s41467-019-08724-x 30770821 PMC6377658

[88] Goncheva MI, Flannagan RS, Heinrichs DE. 2020. De Novo purine biosynthesis is required for intracellular growth of Staphylococcus aureus and for the hypervirulence phenotype of a pure mutant. Infect Immun 88:e00104-20. doi:10.1128/IAI.00104-20 32094249 PMC7171247

[89] Hoiseth SK, Stocker BAD. 1981. Aromatic-dependent salmonella typhimurium are non-virulent and effective as live vaccines. Nature New Biol 291:238–239. doi:10.1038/291238a0 7015147

[90] Ivánovics G, Marjai E, Dobozy A. 1968. The growth of purine mutants of bacillus anthracis in the body of the mouse. J Gen Microbiol 53:147–162. doi:10.1099/00221287-53-2-147 4976582

[91] Jenkins A, Cote C, Twenhafel N, Merkel T, Bozue J, Welkos S. 2011. Role of purine biosynthesis in bacillus anthracis pathogenesis and virulence. Infect Immun 79:153–166. doi:10.1128/IAI.00925-10 21041498 PMC3019915

[92] Mei JM, Nourbakhsh F, Ford CW, Holden DW. 1997. Identification of Staphylococcus aureus virulence genes in a murine model of bacteraemia using signature-tagged mutagenesis. Mol Microbiol 26:399–407. doi:10.1046/j.1365-2958.1997.5911966.x 9383163

[93] Polissi A, Pontiggia A, Feger G, Altieri M, Mottl H, Ferrari L, Simon D. 1998. Large-scale identification of virulence genes from streptococcus pneumoniae. Infect Immun 66:5620–5629. doi:10.1128/IAI.66.12.5620-5629.1998 9826334 PMC108710

[94] Atkins T, Prior RG, Mack K, Russell P, Nelson M, Oyston PCF, Dougan G, Titball RW. 2002. A mutant of Burkholderia pseudomallei, auxotrophic in the branched chain amino acid biosynthetic pathway, is attenuated and protective in A murine model of melioidosis. Infect Immun 70:5290–5294. doi:10.1128/IAI.70.9.5290-5294.2002 12183585 PMC128252

[95] Zhang YJ, Rubin EJ. 2013. Feast or famine: the host-pathogen battle over amino acids. Cell Microbiol 15:1079–1087. doi:10.1111/cmi.12140 23521858 PMC6434321

[96] Zhang G, Ross CR, Blecha F. 2000. Porcine antimicrobial peptides: new prospects for ancient molecules of host defense. Vet Res 31:277–296. doi:10.1051/vetres:2000121 10863946

[97] Cytryńska M, Mak P, Zdybicka-Barabas A, Suder P, Jakubowicz T. 2007. Purification and characterization of eight peptides from galleria mellonella immune hemolymph. Peptides 28:533–546. doi:10.1016/j.peptides.2006.11.010 17194500

[98] Inhülsen S, Aguilar C, Schmid N, Suppiger A, Riedel K, Eberl L. 2012. Identification of functions linking quorum sensing with biofilm formation in Burkholderia cenocepacia H111. Microbiologyopen 1:225–242. doi:10.1002/mbo3.24 22950027 PMC3426421

[99] Butt AT, Thomas MS. 2017. Iron acquisition mechanisms and their role in the irvulence of Burkholderia species. Front Cell Infect Microbiol 7:460. doi:10.3389/fcimb.2017.00460 29164069 PMC5681537

[100] Sousa SA, Feliciano JR, Pita T, Guerreiro SI, Leitão JH. 2017. Burkholderia cepacia complex regulation of virulence gene expression: a review. Genes (Basel) 8:43. doi:10.3390/genes8010043 28106859 PMC5295037

[101] Visser MB, Majumdar S, Hani E, Sokol PA. 2004. Importance of the ornibactin and pyochelin siderophore transport systems in Burkholderia cenocepacia lung infections. Infect Immun 72:2850–2857. doi:10.1128/IAI.72.5.2850-2857.2004 15102796 PMC387874

[102] Darling P, Chan M, Cox AD, Sokol PA. 1998. Siderophore production by cystic fibrosis isolates of Burkholderia cepacia. Infect Immun 66:874–877. doi:10.1128/IAI.66.2.874-877.1998 9453660 PMC107988

[103] Sokol PA. 1986. Production and utilization of pyochelin by clinical isolates of Pseudomonas cepacia. J Clin Microbiol 23:560–562. doi:10.1128/jcm.23.3.560-562.1986 2937804 PMC268694

[104] Mathew A, Jenul C, Carlier AL, Eberl L. 2016. The role of siderophores in metal homeostasis of members of the genus Burkholderia. Environ Microbiol Rep 8:103–109. doi:10.1111/1758-2229.12357 26621188

[105] Sathe S, Mathew A, Agnoli K, Eberl L, Kümmerli R. 2019. Genetic architecture constrains exploitation of siderophore cooperation in the bacterium Burkholderia cenocepacia Evol Lett 3:610–622. doi:10.1002/evl3.144 31844554 PMC6906993

[106] Whitfield C, Trent MS. 2014. Biosynthesis and export of bacterial lipopolysaccharides. Annu Rev Biochem 83:99–128. doi:10.1146/annurev-biochem-060713-035600 24580642

[107] Pier GB. 2007. Pseudomonas aeruginosa lipopolysaccharide: a major virulence factor, initiator of inflammation and target for effective immunity. Int J Med Microbiol 297:277–295. doi:10.1016/j.ijmm.2007.03.012 17466590 PMC1994162

[108] Simpson BW, Trent MS. 2019. Pushing the envelope: LPS modifications and their consequences. Nat Rev Microbiol 17:403–416. doi:10.1038/s41579-019-0201-x 31142822 PMC6913091

[109] Bender JK, Wille T, Blank K, Lange A, Gerlach RG. 2013. LPS structure and PhoQ activity are important for salmonella typhimurium virulence in the galleria mellonella infection model [corrected]. PLoS One 8:e73287. doi:10.1371/journal.pone.0073287 23951347 PMC3738532

