# Supplementary material for: Unraveling Burkholderia cenocepacia H111 fitness determinants using two animal models
Source: mSystems. 2025 Mar 19;10(4):e01354-24. doi: 10.1128/msystems.01354-24 (PMC12013268; doi:10.1128/msystems.01354-24)
Supplement: Supplemental figures — Fig. S1 to S3. [file msystems.01354-24-s0001.pdf]

Supplementary material

Unravelling *Burkholderia cenocepacia* H111 fitness determinants using two animal models

Sarah Paszti[a*], Stefano Gualdi[a*], Marta Torres[a#], Luis Augusto[b], Freya Harrison[c], Leo Eberl[a#]

[a]*Department of Plant and Microbial Biology, University of Zurich, Zürich, Switzerland.*
[b]*Institute for Integrative Biology of the Cell (I2BC), University Paris-Saclay, CEA, CNRS, Gif-sur-Yvette, France.*
[c]*School of Life Sciences, Gibbet Hill Campus, University of Warwick, Coventry, United Kingdom.*
[*]Sarah Paszti and Stefano Gualdi contributed equally to this work
[#]Corresponding authors: marta.torres@botinst.uzh.ch, leberl@botinst.uzh.ch

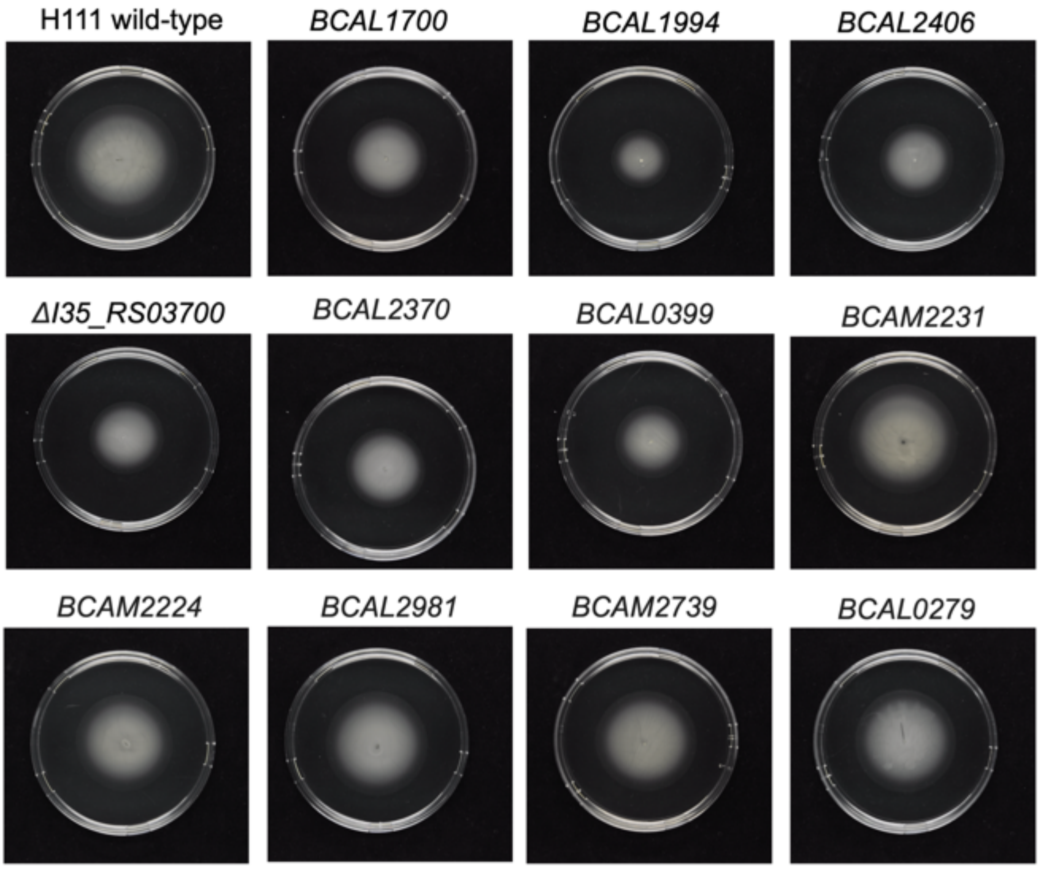

Figure S1. Swimming motility of *B. cenocepacia* H111 and different mutants. Phenotype was evaluated in LB media with 0.2% (w/v) agar after incubation at 37ºC for 48 h.

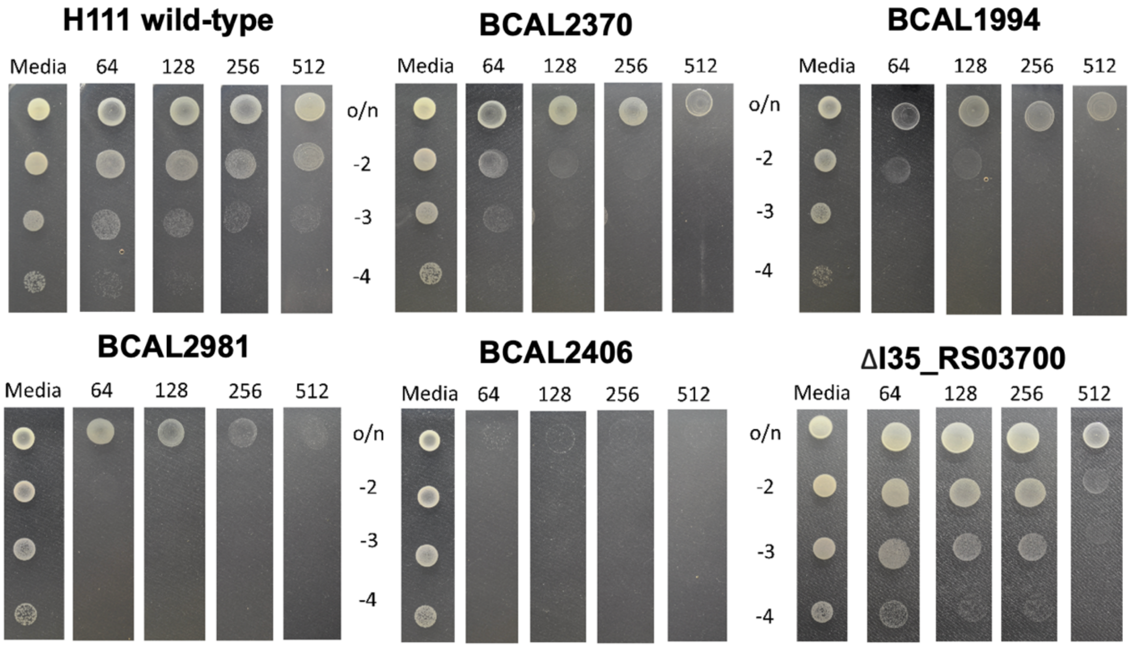

Figure S2. Polymyxin sensitivity test of *B. cenocepacia* H111 and a selection of mutants. Phenotype was evaluated in Müller Hinton II cation adjusted agar medium supplemented with 64, 128, 256 and 512 µg ml-^{1} of polymyxin B sulfate.

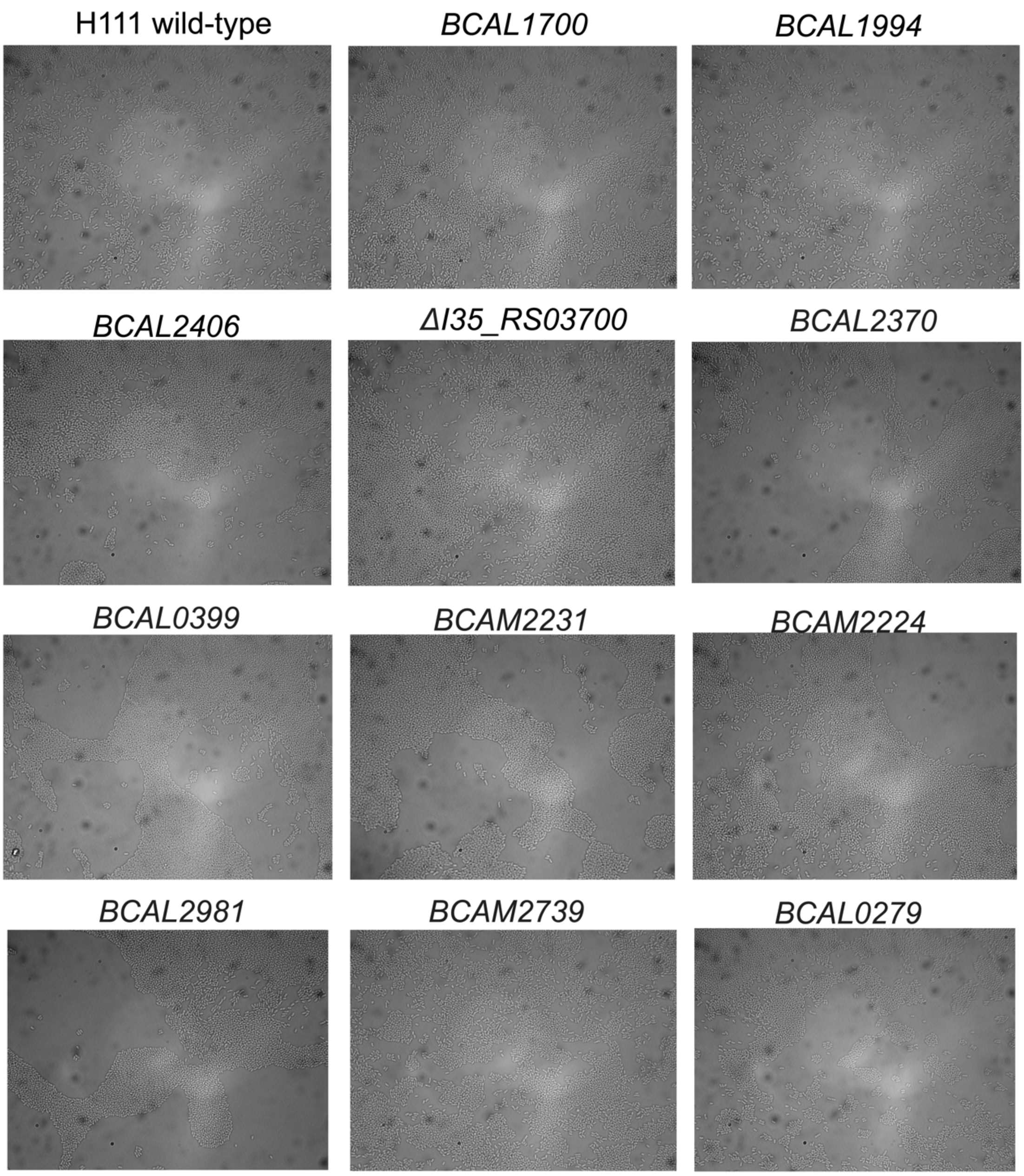

Figure S3. Microscopic observation of *B. cenocepacia* H111 and mutants.
